# Extracellular RNAs-TLR3 signaling contributes to cognitive impairment after chronic neuropathic pain in mice

**DOI:** 10.1038/s41392-023-01543-z

**Published:** 2023-08-07

**Authors:** Xueying Zhang, Rui Gao, Changteng Zhang, Yi Teng, Hai Chen, Qi Li, Changliang Liu, Jiahui Wu, Liuxing Wei, Liyun Deng, Lining Wu, Shixin Ye-Lehmann, Xiaobo Mao, Jin Liu, Tao Zhu, Chan Chen

**Affiliations:** 1 Department of Anesthesiology, West China Hospital, Sichuan University, Chengdu, China; 2 The Research Units of West China (2018RU012)-Chinese Academy of Medical Sciences, West China Hospital, Sichuan University, Chengdu, China; 3 Department of Respiratory and Critical Care Medicine, Targeted Tracer Research and Development Laboratory, West China Hospital, Sichuan University, Chengdu, China; 4 Diseases and Hormones of the Nervous System, University of Paris-Scalay Bicêtre Hosptial, Le Kremlin-Bicêtre, France; 5 Department of Neurology, Institute of Cell Engineering, School of Medicine, Johns Hopkins University, Baltimore, USA

**Keywords:** Regeneration and repair in the nervous system, Neurodevelopmental disorders

## Abstract

Chronic pain is often associated with cognitive decline, which could influence the quality of the patient’s life. Recent studies have suggested that Toll-like receptor 3 (TLR3) is crucial for memory and learning. Nonetheless, the contribution of TLR3 to the pathogenesis of cognitive decline after chronic pain remains unclear. The level of TLR3 in hippocampal neurons increased in the chronic constriction injury (CCI) group than in the sham group in this study. Importantly, compared to the wild-type (WT) mice, TLR3 knockout (KO) mice and TLR3-specific neuronal knockdown mice both displayed improved cognitive function, reduced levels of inflammatory cytokines and neuronal apoptosis and attenuated injury to hippocampal neuroplasticity. Notably, extracellular RNAs (exRNAs), specifically double-stranded RNAs (dsRNAs), were increased in the sciatic nerve, serum, and hippocampus after CCI. The co-localization of dsRNA with TLR3 was also increased in hippocampal neurons. And the administration of poly (I:C), a dsRNA analog, elevated the levels of dsRNAs and TLR3 in the hippocampus, exacerbating hippocampus-dependent memory. In additon, the dsRNA/TLR3 inhibitor improved cognitive function after CCI. Together, our findings suggested that exRNAs, particularly dsRNAs, that were present in the condition of chronic neuropathic pain, activated TLR3, initiated downstream inflammatory and apoptotic signaling, caused damage to synaptic plasticity, and contributed to the etiology of cognitive impairment after chronic neuropathic pain.

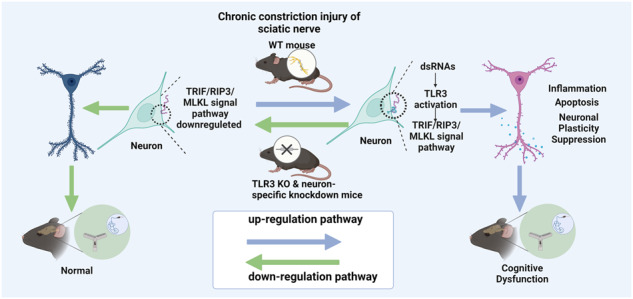

## Introduction

Approximately 11% to 40% of people suffer from chronic pain, according to previous studies.^1,2^ Chronic neuropathic pain is triggered or provoked by the primary injury of nerves and leads to disorders of the central nervous system (CNS) and peripheral nervous system (PNS). People who experience pain frequently ignore it and treat it incorrectly, which has a serious negative effect on their health, capacity for daily activities, and quality of life, imposing heavy economic costs. The typical adverse effects of chronic pain in patients include sleep disturbances, musculoskeletal issues, decreased mobility, falls, malnutrition, cognitive impairment, increased drug use, negative mood, and limited social networking. Meanwhile, changes in the CNS and PNS are frequently linked to chronic pain, including chronic neuropathic pain.^3^ Cognitive impairment induced by chronic pain obviously decreases the quality of life in many ways. Numerous negative effects of chronic pain on cognition, including processing speed, psychomotor speed, executive function, and overall cognitive performance, have been demonstrated by researchers.^4–6^ However, the mechanisms of cognitive impairments after chronic pain are incredibly intricate. Therefore, it is very important to find the critical regulatory factors of cognitive dysfunction induced by chronic neuropathic pain and explore the effective therapeutic targets for prevention and treatment.

Cognitive impairment is a complex disease with some clinical manifestations, such as delirium, mild cognitive impairment (MCI), and dementia. Meanwhile, cognitive impairment is characterized by a decline in previously attained levels of cognitive function, including one or more cognitive domains such as language, learning and memory, executive function, attention, perceptual-motor function, and social interaction.^7^ Therefore, cognitive function is a complex network system regulated by many brain regions, such as the hippocampus, prefrontal cortex and striatum, as well as a series of neural cells (for example, neurons, astrocytes and microglia) and complex molecular pathways.^8,9^ Previously, studies have demonstrated that neuroinflammation, nerve cell apoptosis and synaptic plasticity may mediate the occurrence and development of cognitive impairment. Also, increasing experimental and clinical evidence suggests that chronic pain, can trigger systemic and central responses, thus promoting the release of inflammatory mediators or inducing neurotransmitter changes, leading to cognitive decline, which may be one of the key mechanisms of chronic pain complicated with cognitive impairment.

Toll-like receptors (TLRs), one of the most important defense receptors in the innate nonspecific immune system, have been shown to be capable of recognizing different kinds of damage/danger-associated molecular patterns (DAMPs) and pathogen-associated molecular patterns (PAMPs), as well as activating NF-κB and other transcription factors to trigger the proinflammatory immune response.^10–12^ Then, proinflammatory cytokines could result in neuroinflammation of the CNS and cognitive impairment.^13^ In neurons, TLR3 is a prominent TLR that is expressed at a high level in response to inflammatory stimuli, such as viral infections.^14,15^ Some studies have demonstrated that TLR3 is linked to a variety of cognitive behavioral disorders, including postoperative cognitive decline (POCD), learning and memory dysfunction, Alzheimer’s disease (AD)-related disorders, and schizophrenia-related diseases.^16–19^ Additionally, TLR3 has been shown to be crucial in the management of chronic pain.^20^ According to a study, activation of TLRs could induce inflammatory effects on many neural cells, including microglia and astrocytes, neurons, and other types of cells, thus affecting the process of nociception and causing pain.^16^ Nonetheless, few studies have examined whether TLR3 could be activated in the presence of chronic pain and contribute to the impairment of cognitive function. Identifying the probable function and mechanism of TLR3 in the cognitive decline caused by chronic pain is therefore of great interest.

Double-stranded RNA (dsRNA) is a specific type of extracellular RNA (exRNA) that has garnered significant interest in the field of molecular biology. Previous studies have demonstrated that exRNAs derived from necrotic cells possess the remarkable ability to elicit an inflammatory reaction by upregulating various proinflammatory mediators, as evidenced by the research findings.^21^ Otherwise, the perioperative administration of RNase, an enzyme that degrades RNA molecules, can improve the cognitive dysfunction caused by unilateral nephrectomy in aged mice.^22^ Moreover, emerging researches have shed light on the involvement of dsRNAs in driving the acute inflammatory cascade observed in lung contusions and allergic respiratory diseases. Specifically, it has been proposed that dsRNAs derived from necrotic and damaged cells can activate TLR3, thereby triggering a series of events leading to the initiation and progression of the inflammatory response.^23,24^ These findings provide important insights into the underlying mechanisms of these respiratory conditions and highlight the potential targeting of dsRNAs or TLR3 as a therapeutic strategy. In recent years, studies have also explored the impact of poly (I:C), a synthetic analog of dsRNAs, on the brain’s cytokine responses in aged animals.^25^ Therefore, we conducted this study to better understand how exRNAs, particularly dsRNAs, which were exposed under chronic neuropathic pain-induced stress conditions and triggered TLR3 in the hippocampus and activated downstream signaling, contributed significantly to the development of chronic neuropathic pain-induced cognitive impairment.

## Results

### Chronic constriction injury results in neuropathic pain with cognitive decline in mice

**Fig. 1:**
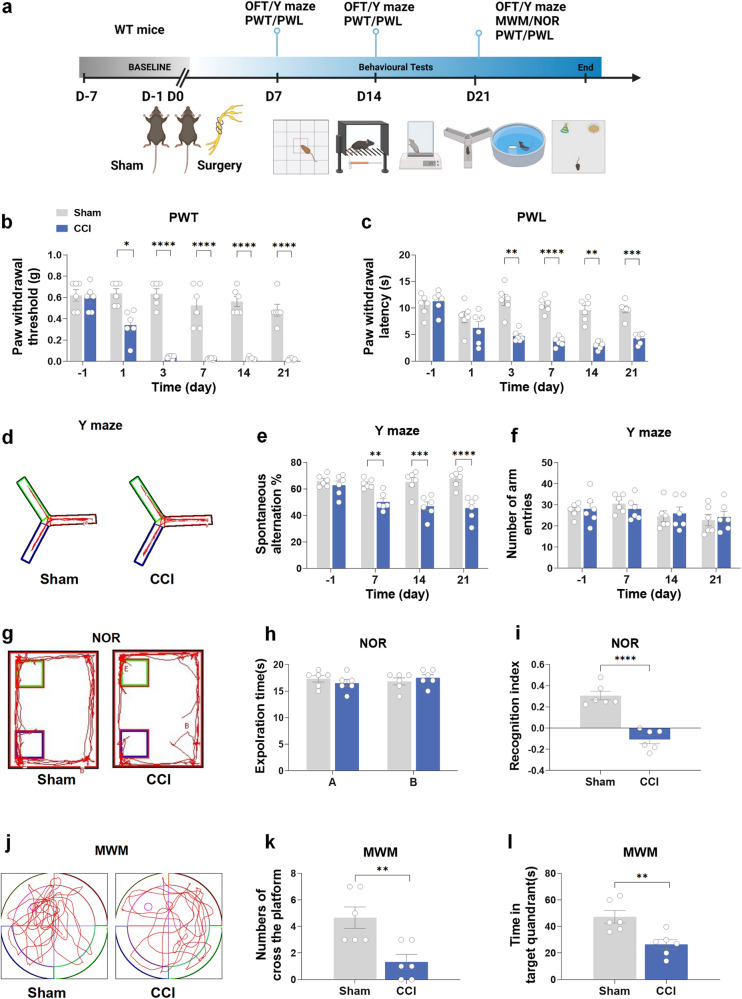
Chronic constriction injury results in neuropathic pain and cognitive decline in mice. **a** Flowchart of this experiment. **b**, **c** The comparison results of PWT and PWL between the CCI group and sham group. **d**–**f** The comparison results of the number of arm entries and the positive spontaneous alternation rate between the sham and CCI groups in the Y-maze test. **g**–**i** The comparison results of the investigation time of objects and the discrimination index between the sham and CCI groups in the NOR test. **j**–**l** Comparison of the spending time of the targeted zone and the number of platform crossings between the sham and CCI groups in the MWM test. CCI chronic constriction injury, OFT open-field test, PWL paw withdrawal latency, PWT paw withdrawal threshold, MWM Morris water maze, NOR novel object recognition. Data are presented as the mean ± SEM (*n* = 6 per group). ^*^P < 0.05, ^**^P < 0.01, ^***^P < 0.001, ^****^P < 0.0001

The flowchart of this experiment is shown in Fig. 1a, and the behavioral tests in this part used the same batch of mice. Initially, we assessed the nociceptive thresholds and cognitive abilities in WT mice after CCI. During the tests that provoked pain, the mice in the CCI group exhibited significant decreases in both the paw withdrawal threshold (PWT) and paw withdrawal latency (PWL) compared to those in the sham group at 3, 7, 14, and 21 days after CCI (Fig. 1b, c). The results of the open-field test (OFT) demonstrated that there was no difference between the sham and CCI groups in the locomotor function of mice (Supplementary Fig. 1a). The Y-maze test verified a significant decrease in spontaneous alternation at both 14 and 21 days after CCI compared to the sham group. However, this decrease was not observed at the 7-day time point (Fig. 1d, e). No significant difference was observed in the total arm entry numbers in the Y-maze between the two groups (Fig. 1f). In the novel object recognition (NOR) test, the reduction in the discrimination index was obvious at 21 days in the CCI group compared to the sham group (Fig. 1g–i). Additionally, during the Morris water maze (MWM) test, mice in the CCI group exhibited decreased time spent in the target quadrant and fewer crossings of the platform in comparison to the sham group (Fig. 1j–l).

### The expression of TLR3 is upregulated in the hippocampus after chronic neuropathic pain

**Fig. 2:**
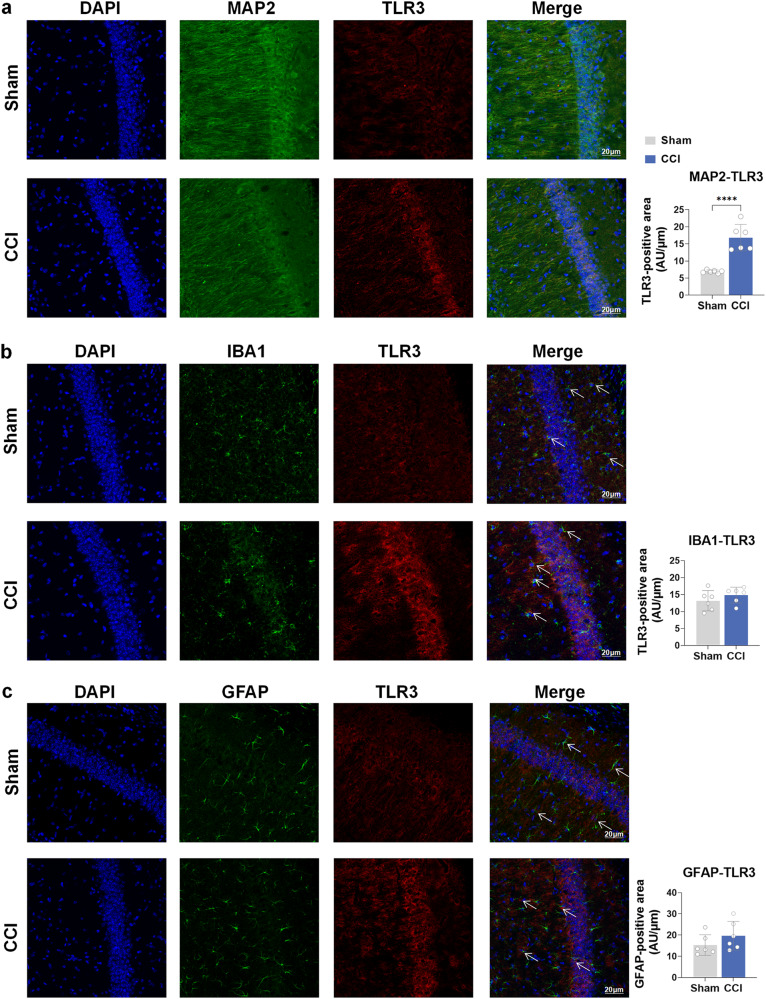
TLR3 is increased in the neurons of the hippocampus in CCI mice as determined by immunofluorescence staining. **a** The colocalization of TLR3 with neurons (MAP2+). Scale bar = 20 μm. **b** The colocalization of TLR3 with microglia (IBA1+). Scale bar = 20 μm. **c** The colocalization of TLR3 with astroglia (GFAP+). Scale bar = 20 μm. Arrows were used to indicate the merge of TLR3 and IBA1 or GFAP. +, Positive expression of the antibody. Data are presented as the mean ± SEM (*n* = 6 per group). ^****^P < 0.0001

To confirm the role of TLR3 in the process of cognitive impairment after CCI, immunofluorescence staining revealed the level of TLR3 at 21 days following CCI or sham surgery. In the CCI group, there was a notable increase in the levels of TLR3 in the CA1, CA3, and DG regions of both the ipsilateral and contralateral hippocampus compared to the sham group (Supplementary Fig. 1a–d). Immunofluorescence staining results indicated a significant upregulation of TLR3 specifically in neurons, while no noticeable increase was observed in microglia and astrocytes (Fig. 2a–c). Based on our observations, we propose that TLR3 plays a vital part in cognitive impairment after CCI.

### TLR3 KO improves the cognitive decline induced by chronic neuropathic pain in mice

**Fig. 3:**
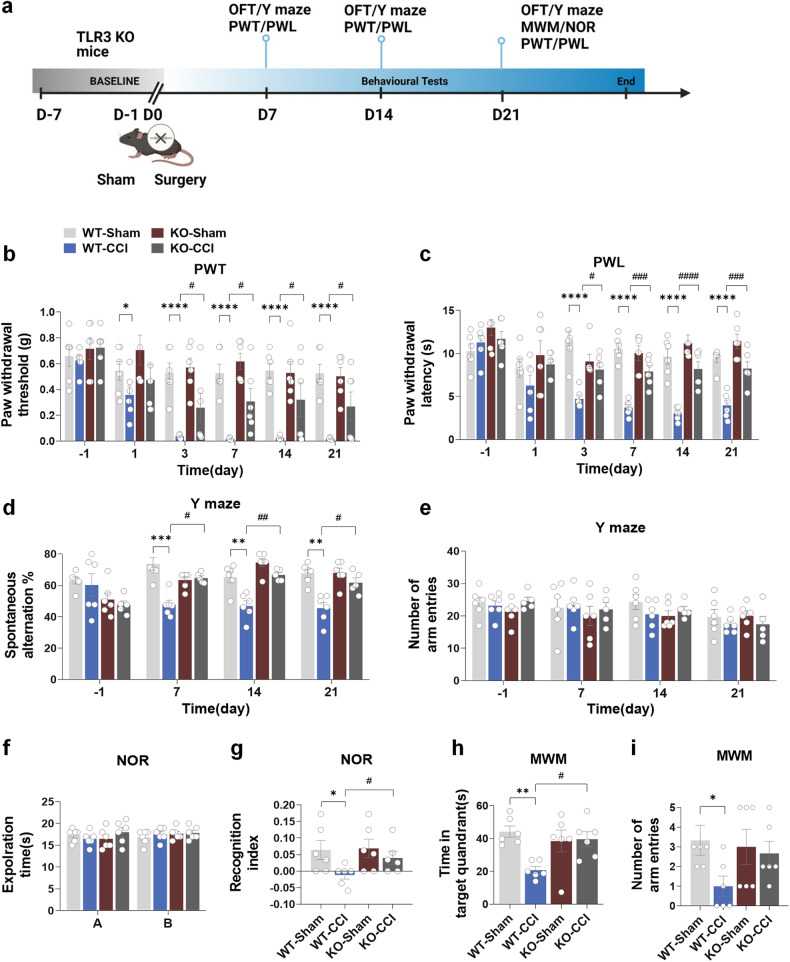
TLR3 KO improves the cognitive decline induced by chronic neuropathic pain in mice. **a** Flowchart of this experiment. **b**, **c** The comparison results of the number of arm entries and spontaneous alternation between four groups in the Y-maze test. **d**, **e** The comparison results of the number of arm entries and the positive spontaneous alternation rate between the four groups in the Y-maze test. **f**, **g** The comparison results of the investigation time of objects and the discrimination index between the four groups in the NOR test. **h**, **i** In the MWM test, the time spent in the target quadrant and the number of platform crossings on the testing day were recorded. Data are presented as the mean ± SEM (*n* = 6 per group). ^*^, WT-Sham vs. WT-CCI; ^#^, WT-CCI vs. KO-CCI. ^*^P < 0.05, ^**^P < 0.01, ^***^P < 0.001, ^****^P < 0.0001; ^#^*P* < 0.05, ^##^*P* < 0.01, ^###^*P* < 0.001, ^####^*P* < 0^.^0001

We proceeded to investigate the impact of TLR3 KO on nociceptive hypersensitivity and cognitive function in mice after CCI. The flowchart of this experiment is shown in Fig. 3a, and the behavioral testing in this part used the same batch of mice of WT mice or TLR3 KO mice. In the TLR3 KO group, notable alleviation of the CCI-induced decreases in paw PWT and PWL was observed (Fig. 3b, c). Furthermore, the results of the Y-maze test showed that TLR3 KO significantly decreased spontaneous alternation in the KO-CCI group compared to the WT-CCI group (Fig. 3d), as well as no significant differences were observed in the total number of arm entries among the four groups (Fig. 3e). As anticipated, no significant disparities in locomotor function were observed among the four groups in the OFT results (Supplementary Fig. 1b). During the training session of the NOR test, the time spent exploring two identical objects was comparable across the four groups (Fig. 3f). However, in the test session, a noteworthy enhancement in the discrimination index was observed in the KO-CCI group in comparison to the WT-CCI group (Fig. 3g). Moreover, in the MWM test, the KO-CCI group exhibited increased time spent in the target quadrant and a higher number of platform crossings than the WT-CCI group (Fig. 3h, i).

### TLR3 KO decreases the inflammatory response and apoptosis in the hippocampus induced by chronic neuropathic pain in mice

**Fig. 4:**
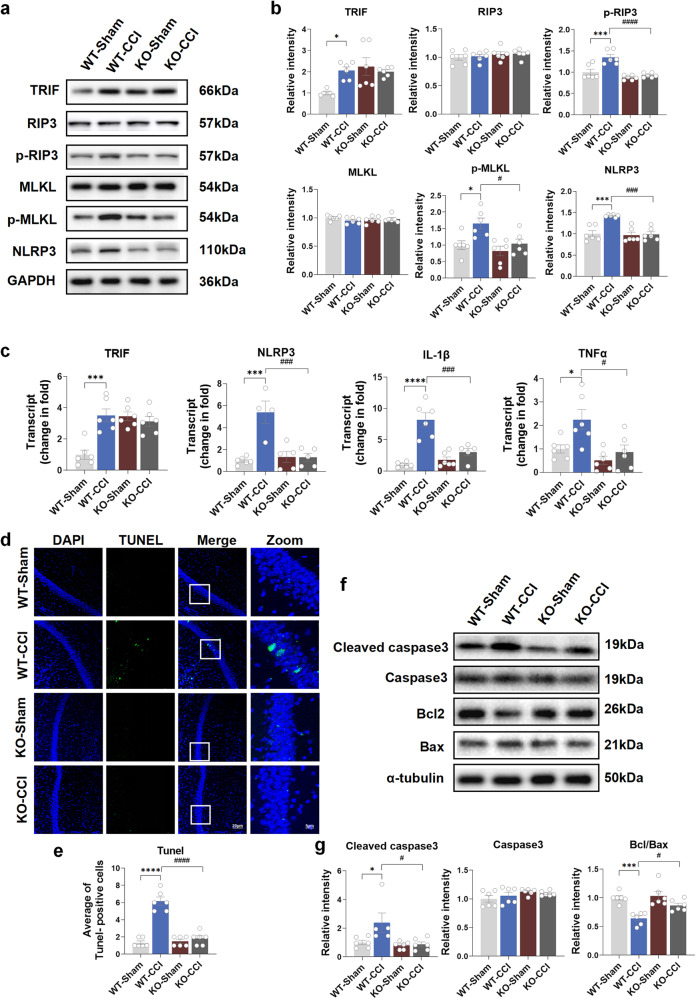
TLR3 KO decreases the inflammatory response and apoptosis in the hippocampus induced by chronic neuropathic pain in mice. **a**, **b** The protein expression of TRIF, RIP3, p-RIP3, MLKL, p-MLKL and NLRP3 in the hippocampus on day 21 after sham surgery and CCI. **c** The mRNA expression of TRIF, NLRP3, IL-1β and TNFα. **d**, **e** TUNEL staining results of the hippocampus 21 days after sham or CCI surgery. Scale bar = 20 μm (DAPI, TUNEL and Merge). Scale bar = 5 μm (Zoom). **f**, **g** The expression of cleaved caspase3, caspase3, bcl2 and bax in the hippocampus of mice 21 days after sham surgery or CCI. Data are presented as the mean ± SEM (*n* = 6 per group). ^*^, WT-Sham vs. WT-CCI; ^#^, WT-CCI vs. KO-CCI. ^*^P < 0.05, ^***^P < 0.001, ^****^P < 0.0001; ^#^*P* < 0.05, ^###^*P* < 0.001, ^####^*P* < 0^.^0001

To elucidate the effect of TLR3 on the inflammatory response and apoptosis in the hippocampus induced by chronic neuropathic pain in mice, the TLR3-evoked downstream signal transduction pathway and related molecules were analyzed in both WT and TLR3 KO mice. Of note, elevated levels of TRIF, p-RIP3, p-MLKL, and NLRP3 were observed in the WT-CCI group compared to the WT-Sham group at both the transcriptional and translational levels (Fig. 4a–c). Interestingly, the KO-CCI group showed a significant reduction in the protein and mRNA expression levels of TLR3, p-RIP3, p-MLKL, and NLRP3 compared to the WT-CCI group (Fig. 4a–c). Surprisingly, elevated protein and mRNA expression of TRIF were observed in the two KO groups compared with the WT groups. This suggests a compensatory increase in TRIF due to the absence of TLR3. TUNEL staining showed that the apoptosis of neural cells in the hippocampus was increased in the WT-CCI group compared to the WT-Sham group (Fig. 4d, e). Furthermore, the WT-CCI group exhibited a marked increase in the levels of cleaved caspase-3 and the ratio of Bcl/Bax compared to the WT-Sham group (Fig. 4f, g). Conversely, neural apoptosis was diminished in the KO-CCI group compared to the WT-CCI group. These findings suggest that the activation of TLR3 and the subsequent involvement of inflammatory and apoptotic molecules may contribute to the development of cognitive decline induced by chronic neuropathic pain.

### TLR3 KO improves the impairment of dendritic spines in the hippocampus induced by chronic neuropathic pain in mice

**Fig. 5:**
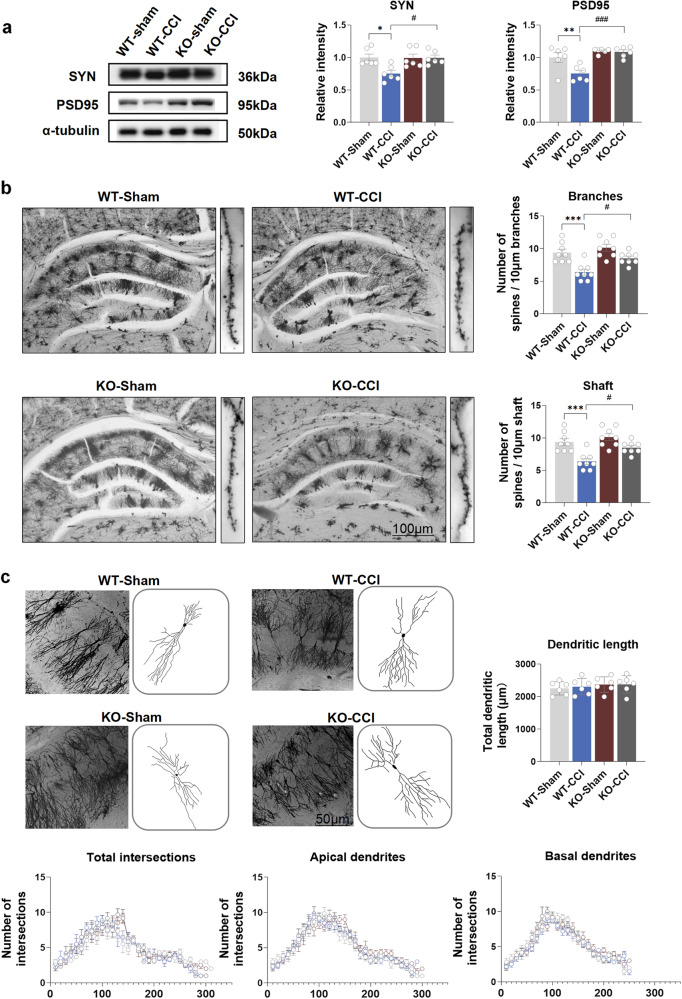
TLR3 KO improves the impairment of dendritic spines in the hippocampus induced by chronic neuropathic pain in mice. **a** The protein levels of PSD95 and SYN in the hippocampus on day 21 after sham surgery and CCI. **b** The results of spine numbers in branches and shafts in CA1 pyramidal neurons on day 21 after sham surgery and CCI. Scale bar = 100 μm. **c** The total dendritic length, total intersection numbers, apical intersection numbers and basal intersection numbers on day 21 after sham surgery or CCI. Scale bar = 50 μm. Data are presented as the mean ± SEM (*n* = 6 per group). ^*^, WT-Sham vs. WT-CCI; ^#^, WT-CCI vs. KO-CCI. ^*^P < 0.05, ^**^P < 0.01, ^***^P < 0.001; ^#^*P* < 0.05, ^###^*P* < 0.001

WB analysis demonstrated an obvious decrease in the protein levels of PSD95 and SYN, which are crucial for neuroplasticity, in the hippocampal tissues of the WT-CCI group compared to the WT-Sham group (Fig. 5a). In contrast, the expressions of PSD95 and SYN in the KO-CCI group were notably higher than those in the WT-CCI group (Fig. 5a). Golgi staining was used to evaluate the status of pyramidal neuronal dendritic spines in the hippocampal CA1 region. The CCI group exhibited a significant reduction in spine density along the neuronal shafts and branches compared to the sham group. These reductions were attenuated in the KO-CCI group (Fig. 5b). However, no significances were observed in either the total dendritic length or intersections among the four groups (Fig. 5c). These findings suggest a crucial role of TLR3 in hippocampal neuroplasticity following chronic neuropathic pain.

### Neuronal-specific knockdown of TLR3 in the hippocampus improves cognitive decline induced by chronic neuropathic pain in mice

To expand our investigation, we proceeded to explore the consequences of neuronal-specific knockdown of TLR3 on nociceptive hypersensitivity and cognitive decline in mice following CCI. The flowchart of this experiment is shown in Supplementary Fig. 3a, and the behavioral testing in this part used the same batch of mice. The model diagram of AAV was showed in Supplementary Fig. 4a. Three weeks after injecting AAV into both hippocampi of mice, Cherry fluorescence was observed in mice injected with AAV, while no fluorescence was observed in control mice (Supplementary Fig. 4b). The results showed that Cherry fluorescence was detected only on neurons rather than microglia and astroglia (Supplementary Fig. 4c). WB analysis revealed an obvious decrease in TLR3 expression in the hippocampus of mice in the AAV^+^ group compared to the AAV^−^ group (Supplementary Fig. S4d, e). No differences were observed in locomotor function among the four groups in the OFT (Supplementary Fig. S1b). Moreover, there were no differences in PWT and PWL between the CCI+AAV^−^ group and CCI+AAV^+^ group (Supplementary Fig. S3b, c). In the Y-maze test, neuronal-specific knockdown of TLR3 led to a noteworthy increase in spontaneous alternation in the CCI+AAV^+^ group compared to the CCI+AAV^−^ group (Supplementary Fig. S3d). There were no differences in the total number of arm entries among the four groups (Supplementary Fig. S3e). In the NOR training period, the exploration time of the two objects was similar across the four groups (Supplementary Fig. S3f). However, in the test session, there was a significant improvement in the discrimination index in the CCI+AAV^+^ group compared to the CCI+AAV^−^ group (Supplementary Fig. S3g). Finally, in the MWM test, the total spending time of the target quadrant and the number of crossings were increased in the CCI+AAV^+^ group compared to the CCI+AAV^−^ group (Supplementary Fig. S3h, i). The WB results also revealed decreased expression of TLR3 and TRIF in the hippocampus of the CCI+AAV^+^ group compared to the CCI+AAV^−^ group (Supplementary Fig. S3j–l).

### Administration of dsRNA analog exacerbates cognitive decline induced by chronic neuropathic pain in mice

To validate the levels of exRNAs following CCI in vivo, we assessed the exRNA levels in the hippocampus, sciatic nerves, and serum. Interestingly, we observed a significant increase in the levels of exRNAs in the hippocampus, sciatic nerves, and serum of the CCI group rather than those of the sham group (Supplementary Fig. 5a). These findings suggest that CCI may trigger an amplified release of exRNAs, subsequently elevating the levels of exRNAs in the bloodstream. Notably, we observed a pronounced increase in the levels of dsRNAs in the sciatic nerves and the hippocampus of the WT-CCI group compared to the WT-Sham group (Supplementary Fig. S5b). In summary, based on our mouse model of CCI-induced cognitive decline, we postulate that increased exRNAs, particularly dsRNAs, might be released from the sciatic nerve into the bloodstream, subsequently leading to elevated levels of dsRNAs in the hippocampus and the activation of TLR3 after chronic neuropathic pain.

**Fig. 6:**
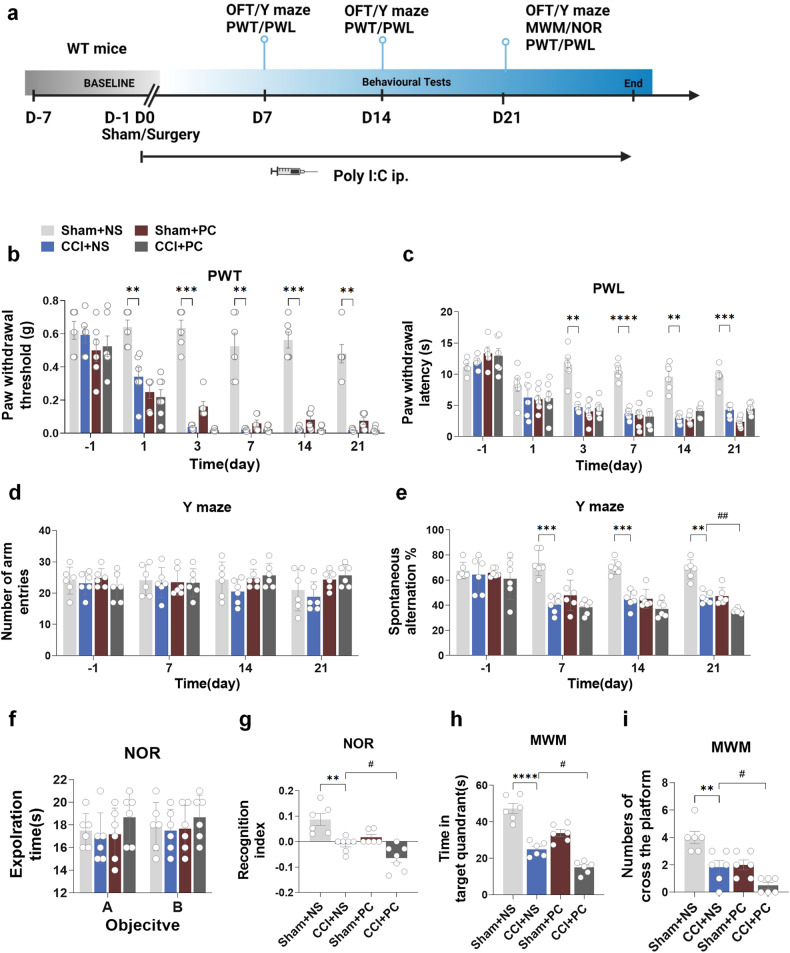
Administration of dsRNA analog exacerbates cognitive decline induced by chronic neuropathic pain in mice. **a** Flowchart of this experiment. **b**, **c** The results of PWT and PWL. **d**, **e** The results of the number of arm entries and the positive spontaneous alternation rate in the Y-maze test. **f**, **g** The results of the investigation time of objects and the discrimination index in the NOR test. **h**, **i** Time spent in the target quadrant and the number of platform crossings in the MWM test. Data are presented as the mean ± SEM (*n* = 6 per group). ^*^, Sham+NS. vs. CC + NS. ; ^#^, CCI+PC vs. CCI+PC. NS, normal saline; PC, poly (I:C). ^*^P < 0.05, ^**^P < 0.01, ^***^P < 0.001, ^****^P < 0.0001; ^#^*P* < 0.05, ^##^*P* < 0.01, ^###^*P* < 0.001, ^####^*P* < 0^.^0001

Thus, we further determined whether poly (I:C), an analog of dsRNA, could aggravate this effect. Mice in the PC group were injected with poly (I:C) 2 μg/g (concentration 1 μg/μL) intraperitoneally 30 min before surgery, during surgery (after sciatic nerve exposure), 1 h after surgery, and 7, 14 and 21 days after surgery. The flowchart of this experiment is shown in Fig. 6a. The injection of poly (I:C) did not affect the locomotor ability of all groups (Supplementary Fig. 1c). Interestingly, multiple intraperitoneal injections of poly (I:C) significantly reduced both PWT and PWL between the Sham+NS group and Sham+PC group, but no changes in PWT and PWL were observed between the CCI+NS and CCI+PC groups. (Fig. 6b, c). The spontaneous ratio of the Y-maze was decreased in the CCI+PC group compared with the CCI+NS group, while there was no difference in the number of arm entries between the four groups (Fig. 6d, e). The CCI+PC group exhibited a decreased discrimination index in the NOR test compared to the CCI+NS group, while there were no differences in exploration time among the four groups (Fig. 6f, g). Furthermore, in the MWM test, the CCI+PC group displayed reduced time spent in the target quadrant and fewer platform crossings than the CCI+NS group (Fig. 6h, i). These findings indicate that the dsRNA analog poly (I:C) could exacerbate cognitive decline following CCI. To gain further insights into the downstream signaling pathway involved in TLR3 activation by poly (I:C), we analyzed the protein expression levels of these downstream molecules. The WB results of the hippocampus revealed upregulation in the expression of TLR3, TRIF, p-RIP3, p-MLKL, and NLRP3 in the CCI+PC group compared to the CCI+NS group (Supplementary Fig. 6a, b). Poly (I:C) also downregulated SYN and PSD95 expression in the hippocampus (Supplementary Fig. 6c). Meanwhile, poly (I:C) activated the cleaved caspase3/caspase3 apoptosis pathway (Supplementary Fig. 6d). Thus, these data indicate that the dsRNA analog could mediate the pathogenesis of cognitive decline after CCI by activating TLR3 and downstream inflammatory and apoptotic molecules.

Furthermore, we also detected the influence of poly I: C on TLR3 KO mice. The flowchart of this experiment is shown in Supplementary Fig. 7a. The OFT results indicated that poly I: C did not influence the total distance between groups (Supplementary Fig. 1d). In the pain threshold tests, there were no differences in the CCI+PC+KO group compared to the CCI+NS+KO group (Supplementary Fig. 7b, c). In the Y-maze test, the spontaneous rate was not affected by poly I: C in the CCI+PC+KO group compared with the CCI+NS+KO group (Supplementary Fig. 7d, e). In the NOR test, there was no difference in the recognition rate between the CCI+PC+KO group and the CCI+NS+KO group (Supplementary Fig. 7f, g). In the MWM test, there were no differences observed in the spending time of the targeted zones and the number of crossings between the CCI+PC+KO group and CCI+NS+KO group (Supplementary Fig. S7h, i). Therefore, poly (I:C) did not work in TLR3 KO mice. We also detected the protein expression levels of the downstream molecules of TLR3. WB results of the hippocampus showed that there were no differences in the expression of TRIF, p-RIP3, p-MLKL, and NLRP3 between the CCI+PC+KO group and CCI+NS+KO group (Supplementary Fig. 8a–g). Poly (I:C) also did not affect SYN and PSD95 expression in the hippocampus of TLR3 KO mice (Supplementary Fig. 8h, j, k). Meanwhile, the cleaved caspase3/caspase3 ratio was not affected (Supplementary Fig. 8i, l, m). Thus, these data indicate that the dsRNA analog could not work in TLR3 KO mice.

### Administration of dsRNA/TLR3 inhibitor improves cognitive decline induced by chronic neuropathic pain in mice

We used dsRNA/TLR3 inhibitor to study the effects of blocking the dsRNA/TLR3 pathway on chronic pain-induced cognitive decline. The flowchart of this experiment is shown in Supplementary Fig. 9a. Mice in the INH group were injected with dsRNA/TLR3 complex inhibitor 30 μg/g (concentration 30 μg/μl) intraperitoneally 30 min before surgery, 1 h after surgery (after sciatic nerve exposure), and 7, 14, and 21 days after surgery. The behavioral testing in this part used the same batch of mice. The injection of dsRNA/TLR3 inhibitor did not affect the locomotor ability of all groups (Supplementary Fig. 1f). dsRNA/TLR3 inhibitor multiple intraperitoneal injections significantly improved the nociceptive thresholds of both the PWT and PWL at 3, 7, 14, and 21 days in the INH group compared with the NS group after CCI (Supplementary Fig. 9b, c). The spontaneous ratio of the Y-maze was increased in the INH group compared with that in the NS group after CCI, while there was no difference in the number of arm entries between the four groups (Supplementary Fig. 9d, e). The discrimination index of NOR was also increased in the CCI+INH group compared with that in the CCI+NS group. group, while there was no difference in the exploration time between the four groups (Supplementary Fig. 9f, g). Meanwhile, the spending time of the targeted zones and the number of crossings in the MWM test were increased in the CCI+INH group compared with the CCI+NS group (Supplementary Fig. 9h, i). Thus, the dsRNA/TLR3 inhibitor could improve cognitive decline after CCI.

We also detected the expression of dsRNAs and TLR3 after treatment with the inhibitor for 21 days. The results showed that the levels of dsRNAs were all decreased in the sciatic nerves, hippocampus and serum after multiple intraperitoneal injections of the dsRNA/TLR3 inhibitor (Supplementary Fig. 10a). The WB results showed that TLR3 was decreased in the hippocampus (Supplementary Fig. 10b).

### Schwann cell-derived exRNAs/dsRNAs upregulate TLR3 in hippocampal neurons in vitro

**Fig. 7:**
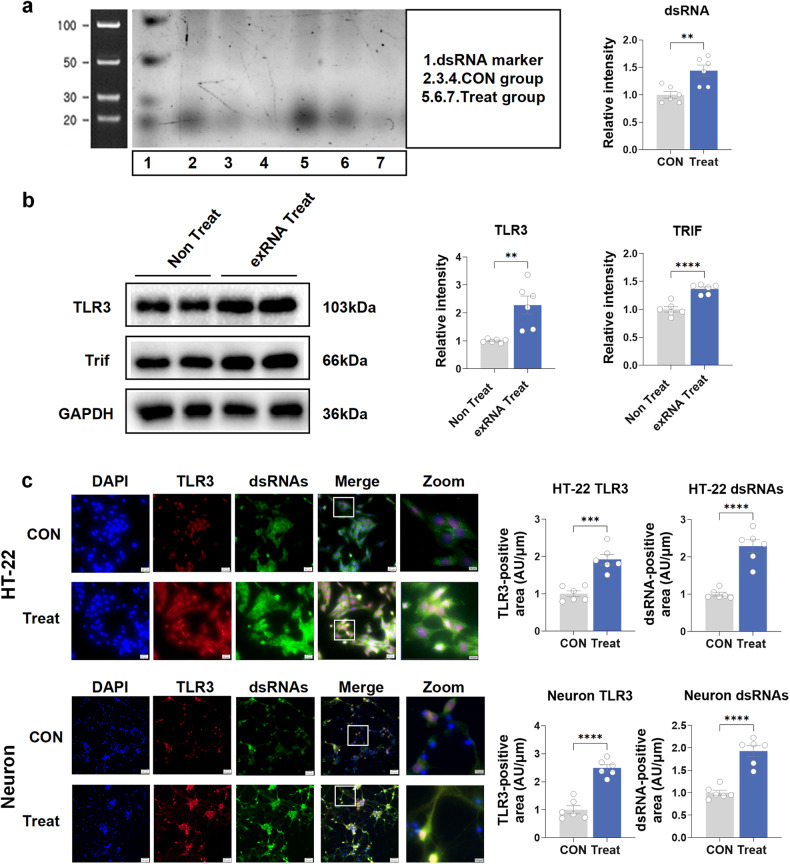
Administration of dsRNA/TLR3 inhibitor improves cognitive decline induced by chronic neuropathic pain in mice. **a** The dsRNA levels of the supernatant total RNAs from treated RSC96 cells by nondenaturing polyacrylamide gel electrophoresis. **b** The effect of exogenous exRNAs/dsRNA derived from RSC96 cells on TLR3 and TRIF protein expression in the HT-22 cell line. **c** The colocalization of TLR3 and dsRNA in the HT-22 cell line and primary cultured neurons after exogenous exRNA treatment. Scale bar = 20 μm (DAPI, TLR3, dsRNAs and Merge). Scale bar = 10 μm (Zoom). Data are presented as the mean ± SEM (*n* = 6 per group). ^*^, CON vs. Treat. CON, control groups without exogenous exRNAs treated; exRNA Treat, group with exogenous exRNAs treated. ^**^P < 0.01, ^***^P < 0.001, ^****^P < 0.001

To determine the reason why peripheral nerve neuropathic pain can cause central nervous system inflammation and other responses leading to cognitive impairment, we hypothesized that after the sciatic nerve was damaged, exRNAs/dsRNAs were released; thus, TLR3 was activated in the hippocampus. The Schwann cell line RSC96 was treated with capsaicin (1 µM) for 48 h and then cultured overnight in a serum-free medium for supernatant total RNA collection. Nondenaturing polyacrylamide gel electrophoresis demonstrated an elevation in dsRNA levels of these supernatant total RNAs from treated RSC96 cells compared with the no-treatment group (Fig. 7a). We stimulated hippocampal neuron HT-22 cells with the RNAs derived from this supernatant. We showed that the levels of TLR3 and TRIF protein were obviously increased in the experimental group stimulated by exogenous RNA compared with the control group (Fig. 7b). Furthermore, fluorescence staining revealed a notable increase in dsRNA levels, along with colocalization with TLR3, in both HT-22 cells and primary cultured neurons following exogenous RNA treatment (Fig. 7c). Meanwhile, exRNAs were also upregulated in primary neurons after RNA treatment (Supplementary Fig. 11a). However, neither exRNAs nor dsRNAs were affected by RNAs from Schwann cells in the KO-Neuron Treat group compared to the KO-Neuron CON group (Supplementary Fig. 11b). Therefore, these results indicated that peripheral sciatic nerve cell-derived exRNAs/dsRNAs could upregulate TLR3 in hippocampal neurons.

## Discussion

Numerous clinical studies have indicated that patients suffering from chronic pain exhibit memory impairment. Researchers have discovered a connection between chronic pain and problems with concentration, recall, executive organizing, and information processing.^26,27^ In addition, the results of our earlier meta-analysis suggested that there may be a connection between the occurrence of cognitive impairment and the presence of chronic pain.^6^ Preclinical studies have also indicated memory and learning deficits in rodent inflammation and neuropathic pain models.^28–30^ The fundamental mechanism of cognitive impairment after chronic pain disorders, however, remains unknown. To address this, we used CCI of the sciatic nerve, a classic chronic neuropathic pain model, to generate cognitive impairment after chronic neuropathic pain in mice. In our study, we made four major findings. First, the CCI group of the WT mice with the reduced nociceptive thresholds demonstrated deficits in working memory, recognition memory, spatial memory, and learning. Second, we observed a significant increase in TLR3 expression in the hippocampal neurons of WT mice with impaired cognitive function after chronic neuropathic pain. Third, either TLR3 KO mice or mice with neuronal-specific knockdown of TLR3 (without affecting the pain threshold) showed enhanced cognitive function compared to WT mice, potentially by mitigating hippocampal inflammation, apoptosis, and impaired neuroplasticity. The downstream signaling pathway driven by TLR3, TRIF/RIP3/MLKL, was demonstrated to be involved in neuroinflammation, neural apoptosis and impaired neuroplasticity. Fourth, the levels of both exRNAs and dsRNAs in the serum, sciatic nerves and hippocampus were increased after CCI. Of note, the artificial synthetic dsRNA, poly (I:C) could exacerbate the cognitive decline induced by CCI through activation of TLR3. Importantly, dsRNA/TLR3 inhibitors could also reverse the cognitive decline after CCI by blocking the binding of dsRNAs and TLR3, suggesting that the binding of dsRNAs and TLR3 was crucial for the progression of cognitive disturbance after chronic neuropathic pain. Taken together, targeting the dsRNA/TLR3 pathway may have potential clinical application prospects for the treatment of chronic pain with cognitive decline. In vitro experiments showed the colocalization of dsRNAs and TLR3 in neurons. Therefore, this study investigated the role of exRNAs, particularly dsRNAs derived from injured sciatic nerves, in activating TLR3 and its associated downstream pathways in vivo and in vitro. The identified mechanisms have been observed to be associated with the cognitive decline observed in chronic neuropathic pain conditions.

TLRs, the first line of defense, have been demonstrated to initiate the proinflammatory immune response by recognizing DAMPs and PAMPs.^10^ Increased evidence has shown that they can also mediate neuroinflammation in the CNS, thus contributing to the development of cognitive impairment.^13^ Of note, TLR3 has been demonstrated to promote neuropathic pain in the rat SNL model.^31^ Additionally, TLR3 can cause varying degrees of dysfunction in the CNS and play an important role in the development of cognitive impairment after anesthesia and surgical operation. To the best of our knowledge, TLR3 is the only TLR that recognizes dsRNAs. Moreover, we found that the mRNA level of TLR3 was the most increased TLR in the hippocampal tissue of our mouse model of neuropathic pain. Therefore, in our current study, we first investigated whether released dsRNAs could trigger the upregulation of TLR3 and contribute to the development of cognitive decline in a mouse model of CCI. Interestingly, we found that dsRNA-TLR3 signaling indeed mediates impaired cognition in a mouse model of neuropathic pain. It has been observed that the level of exRNAs was decreased in a mouse model of unilateral nephrectomy under surgical stress. The specific RNA molecule and the related TLR involved in the mechanism of cognitive impairment associated with chronic pain have yet to be determined.

According to an increasing number of studies, exRNAs could work as a kind of mediators and transmitters between different cells and systems.^32,33^ Cells under necrotic, apoptotic or other stimulated uninfected death conditions can release dsRNAs, a kind of exRNAs, into the extracellular matrix. dsRNA can be recognized by TLR3, thus activating inflammation and apoptosis signaling.^34^ Poly (I:C) has recently been demonstrated to produce increased cytokines in the brains of aged animals. This was related to acute deficiencies in working memory in aged mice.^25^ However, the direct relationship between dsRNA and cognitive decline still needs more exploration. Our study showed that dsRNAs were increased in peripheral nerves, blood circulation and the CNS, so we speculate that under this stress condition, there may be communication from the peripheral to the central nervous system. Indeed, we did not prove this process in animal experiments; however, our cell experiments showed that exRNAs, including dsRNAs derived from Schwann cells, could activate TLR3 in hippocampal neurons. The in vitro results might provide indirect proof of our hypothesis. Then, the clearance of the BBB allows plasma albumin (molecular weight 69,000 KD) to pass through. The size of the dsRNA ranges from tens to hundreds of KD, which can theoretically pass the BBB. At present, there are no methods to trace dsRNAs. However, recent studies have suggested that information and substances such as dsRNAs may be transmitted between the periphery and the center through some carriers, such as some kinds of vesicles.^35,36^ In the future, we plan to explore the tracer of dsRNAs between the CNS and periphery from the carrier of dsRNAs perspective.

Indeed, either injury itself or pain could cause an elevation of the dsRNA levels. Chronic neuropathic pain is characterized by the presence of anomalous sensations known as allodynia, which refers to pain arising from stimuli that are typically nonpainful.^37^ In our mouse model of chronic neuropathic pain, the mouse CCI model, we observed that the injury (original stimuli) had almost recovered, while the pain still lasted on Day 21. Of note, dsRNAs were all detected with increased levels in the sciatic nerves, hippocampus and serum on Day 21, accompanied by impaired cognition, as shown in all the behavior tests, such as the Y-maze, NOR and MWM. Thus, it is strongly suggested that increased levels of dsRNAs on day 21 may be mainly associated with persistent pain-induced cognitive decline. It is worth noting that additional research is required to comprehensively elucidate the differentiation between cognitive decline induced by injury and pain.

Previously, neuroinflammation, apoptosis of neural cells, and synaptic plasticity changes have been demonstrated to be the fundamental mechanisms for developing cognitive disorders in animal models. For detailed mechanisms, the initiation and development of chronic pain-induced cognitive decline is still unclear. Research has demonstrated that TRIF plays a crucial role in the TLR3-mediated signaling pathways that are involved in the protection of mammalian hosts against viral infections.^38^ TRIF can mediate necrosis via RIP3 and MLKL.^39^ Therefore, TLR3 may mediate inflammation through the TRIF/RIP3/MLKL pathway. Moreover, the involvement of the NLRP3 inflammasome has been suggested in various chronic inflammatory disorders,^40^ and its involvement in neurocognitive disorders has also been investigated.^41,42^ In our research, we found that TLR3 KO could reverse inflammation and apoptosis in the hippocampus and change the TRIF/RIP3/MLKL pathway. However, we did not prove again that the downstream pathway of TLR3 leads to inflammation and apoptosis of cells by interfering with the downstream pathway of TLR3. Otherwise, we explored the plasticity of hippocampal neurons, which is important for the maintenance of cognitive function.^43^ PSD95 is a critical scaffolding protein found in the excitatory postsynaptic density. Its primary function is to interact with and regulate various vital molecules, thereby playing a crucial role in neuronal function. Abnormal expression of PSD95 has been documented in various human disorders affecting both the CNS and PNS, such as Alzheimer’s disease, Huntington’s disease, schizophrenia, autism spectrum disease, and pain.^44,45^ SYN encodes phosphoproteins that play crucial roles in the regulation of neurotransmission and neurodevelopment.^46^ The results showed that TLR3 contributed to neuroplasticity changes by reducing dendritic spines in neurons and inhibiting the expression of PSD95 and SYN. However, very few studies have focused on the mechanism by which peripheral nerve injury induces CNS changes.

Schwann cells, initiating the initial response to nerve injury, play a critical part in the mechanism of chronic neuropathic pain. Following the CCI of the sciatic nerve, notable transformations occur in activated Schwann cells, including a shift in phenotype, an increase in proliferation and migration, and the secretion of various factors, such as TNF-α, IL-1, and IL-6.^47^ In our study, we demonstrated that exRNAs derived from injured sciatic nerves play a vital role in the activation of TLR3. In addition, there were some other cells around the sciatic nerves. Macrophages, attracted by Schwann cells, can rapidly arrive at the site of nerve injury.^48^ Studies have shown that macrophages mainly accumulate and infiltrate at the site of nerve injury, thus participating in nerve inflammation and nerve regeneration.^49^ Pericytes are also a cell group that exists near the sciatic nerve, but pericytes are mainly related to nerves and the surrounding blood supply. Studies have shown that pericyte-derived vesicles are involved in nerve regeneration during peripheral nerve injury.^50^ Whether macrophages and pericytes are related to the release of dsRNAs may require further exploration.

Our study has some limitations. In our in vitro experiment, we used capsaicin to stimulate Schwann cells and induce an increase in exRNAs, aiming to mimic the injury caused by CCI. However, it is important to note that capsaicin stimulation does not fully replicate the chronic pain cell model, despite its previous usage in activating Schwann cells in other studies.^51^ Additionally, we put forward that the upregulation of TLR3 in the hippocampus was induced by the increased exRNAs/dsRNAs from activated Schwann cells. However, further investigations are necessary to verify whether the increased exRNAs, especially dsRNAs in the CNS, arise through the BBB directly or indirectly.

In conclusion, the overexpression of TLR3 in mice following CCI could potentially function as an endogenous sensor for exRNAs/dsRNAs. Targeting the dsRNA-TLR3 signaling pathway holds the potential to alleviate hippocampal inflammatory responses, neuronal apoptosis, and neuroplasticity changes, thereby improving cognitive decline under chronic pain conditions. Based on our findings, it appears that exRNAs, specifically dsRNAs, can activate the TLR3 signaling pathway. This process may play a vital part in the development of cognitive impairment in mice who suffer from chronic neuropathic pain. These insights may inspire the exploration of new therapeutic targets aimed at preventing and treating cognitive impairment induced by chronic neuropathic pain.

## Materials and methods

### Animals

TLR3 KO mice were sourced from the Jackson Laboratory (America), while aged-matched C57BL/6 WT male mice weighing 20–25 g and 6–8 weeks old were obtained from Dossy Life Science Company (Chengdu, China). The research was granted clearance by the Animal Care and Use Committee of Sichuan University (NO. 20220519006) and followed the directives specified in the Guide for the Care and Use of Laboratory Animals, as published by the US National Institutes of Health.

### Murine model of chronic neuropathic pain

Chronic neuropathic pain has been induced through CCI surgery as described by Bennet and Xie.^52^ The left trunk of the sciatic nerve was exposed after the anesthetic procedure. At a distance of 1.0–1.5 mm apart, three ligatures were wrapped loosely around the sciatic nerve (5-0 chromic gut; Boda, China). The sciatic nerve on the left was exposed but not ligated in the sham group. Finally, the muscle and skin layers of the incision were sutured.

### Behavioral tests

#### Pain-related behaviors

The mice were given 30 min in Plexiglas chambers with a wire net floor to become accustomed to their new environment. A von Frey filament (Aesthesio, Ugo Basile, Italy) was utilized in order to perform mechanical threshold testing. Filaments of varying weights from 0.008 to 2.0 g were applied orthogonally to the plantar surface, commencing with the minimum force and advancing in an increasing manner. A positive response was deemed to be paw withdrawal, flinching, or paw licking. The “updown” method was used to calculate the 50% PWT. The researchers conducting the tests were blinded to the experimental groups.

The assessment of thermal hyperalgesia was conducted using a thermal testing apparatus (No. 37370, Ugo Basile, Italy) to measure the PWL. The rodents were positioned on a raised glass surface and given a half hour to acclimate before the examination. A thermal stimulator emitting radiant energy was directed toward the planta pedis of the experimental hind paw with the aid of a glass floor. The criterion utilized for a positive reaction was the elevation or licking of the posterior paw, and the duration required to achieve the criterion was documented as the PWL. In order to avoid any potential harm to the tissue, a time limit of 20 s was established.

#### Open-field test

The OFT was tested in a quiet, dimly lit room using a Plexiglas zone (50 × 80 × 30 cm) to assess locomotor activity. To allow mice to adapt to the environment, they were taken to the screening room 1 h prior to the formal test. The total distance of the mice in the test zone was recorded using a video-tracking system (Smart 2.5, Reward, Shenzhen, China) and analyzed for 5 min. After each mouse was tested, the tester cleaned the test box and wiped it with 75% ethanol.

#### Y-maze test

The spontaneous alternation test of the Y-maze measures mice’s preference for visiting a fresh arm of the maze instead of the first arm to assess exploratory behavior. The device was made up of three enclosed arms with a 120° angle. Each mouse underwent a 5-min adaptation period in a quiet room, during which they were gently placed in one arm of the maze. Following the adaptation period, the mouse was placed in another arm for an 8-min test period, and its movements were tracked and analyzed using a tracking system. An observer blinded to the experimental groups scored the sequence and number of arm entries. The mouse was considered spontaneously alternating when it entered all three arms in successive choices (ABC, BCA, or CAB), eliminating repeated inputs (BAB, CAC, or CBC). The percentage of spontaneous alternation was determined as follows: % spontaneous alternation = number of positive spontaneous alternations/(total arm entries − 2) × 100.

#### Novel-object recognition test

The NOR test was conducted in a gray, opaque Plexiglas zone (50 × 80 × 30 cm). On the first day of the experiment, the murine subjects were brought into the enclosure and subjected to a 20-min period of adaptation without any accompanying stimuli. After a duration of 4 h, a pair of indistinguishable items (A and B) were introduced into the enclosure, and mice were permitted to investigate them for a period of 5 min. The duration of exploration for each object was recorded. Following a 24-h retention period, a test was performed in the same box, with one of the familiar objects from the previous session exchanged with a novel object (A and C). The mice were given 5 min to freely explore the objects, and the time spent with each object was recorded. To evaluate cognitive function, the ratio of (C − A)/(A + C) was defined as the recognition index.

#### Morris water maze test

Mice were adapted to the testing room for 1 h before the formal test. The maze consists of a pool with a diameter of 150 cm and a height of 60 cm; the platform to exit is 10 cm in diameter and 28 cm in height. The mice were prohibited from floating float by keeping the water between 22 and 25 °C. The mice underwent training sessions over three consecutive days, with four trials from four different quadrants each day per mouse. If the mouse reached the platform within 120 s, the trial was judged complete. If a mouse had trouble locating the hidden platform during a trial, it was coaxed there. The latency and route taken to reach the platform were recorded and monitored by the tracking system. The function of spatial memory was assessed by a probe test on day 5. The results from the probe trial were analyzed by assessing the total time the mice spent in the primary target quadrant and the number of mice crossing this quadrant.

#### Specimen collection and processing procedures

After anesthetizing the mice, the mouse’s chest cavity was opened, all the blood was extracted from the right ventricle using a sterile injector, and then blood was acquired. The mouse brain tissue was then immediately removed, the surface blood was rinsed in an ice-cold PBS solution, and eventually, the hippocampal tissue was detached. The hippocampal tissues were rapidly frozen in liquid nitrogen and frozen at −80 °C in a refrigerator. After the whole blood collection, it was allowed to stand undisturbed at room temperature (RT) for 15 min. The supernatant, also known as serum, was acquired at 2500 × *g* for 15 min in the centrifuge. These procedures were conducted under strict RNase-free conditions.

#### Immunofluorescence staining

The brains were removed, fixed with the fixed liquid organization at 4 °C for 24 h, and dehydrated in 30% sucrose solution for at least 48 h. Before the brains were sliced, they were buried in tissue-tek OCT solution, frozen, and cut at a thickness of 40 μm from the coronal surface of the brain tissue in the kryotome (Leica CM 3050S). The brain tissue sections underwent three washes with PBS, followed by incubation in a blocking solution. This incubation occurred for 1 h at RT and then stayed overnight at 4 °C. The primary antibodies used were as follows: anti-TLR3 rabbit polyclonal antibody (1:200, ab62566, Abcam), anti-MAP2 chicken antibody (1:200, ab5392, Abcam), anti-NeuN mouse antibody (1:100, 26975, Proteintech), anti-GFAP mouse antibody (1:200, 60190-1-Ig, Proteintech), anti-IBA1 goat antibody (1:100, 011-27991, WAKO) and anti-dsRNA mouse antibody J2 (1:200, 10010500, SCICONS). After overnight incubation with the primary antibodies, the sections were rinsed three times. Then, these sections were incubated in the following secondary antibodies at RT for 1.5 h: Fluor-555 labeled donkey anti-rabbit IgG (1:500, A31572, Invitrogen), Fluor-488 labeled donkey anti-mouse IgG (1:500, A21202, Invitrogen), and Fluor-647 labeled donkey anti-chicken IgY (1:500, ab150175, Abam). In vitro, cells were fixed in 4% formaldehyde for 15 min at RT. Fixed cells were washed in PBS three times and incubated in the blocking solution. Then, the fixed cells were covered with a solution of primary antibodies at 4 °C overnight. After primary antibody incubation overnight, the cells were rinsed in PBS solution three times and covered with secondary antibody solution for 1.5 h at RT. After incubation with secondary antibodies, the sections were stained with DAPI for 5 min (1:1000, ab104139, Abcam). The presence of immunoreactivity was observed using confocal microscopy (Leica SP8, Germany).

#### Primary hippocampal neuron culture

Primary hippocampal neurons were derived from embryos of pregnant TLR3 KO mice or WT mice at 16–17 days, following previously described methods.^53^ The dissociated neurons were seeded on polylysine-covered cell culture dishes in a neurobasal medium containing 5% fetal bovine serum (FBS) (Invitrogen, USA). After 2 h of seeding, the primary cells were cultured in FBS-free neurobasal medium at 37 °C. The cultures were kept in a 5% CO_2_ environment at 37 °C. Half of the medium was changed every 3 days. On the third day, cytarabine (10 μM) was used to inhibit glial cell proliferation. The experiments were conducted using the neurons on day 14.

#### exRNA treatment

The murine hippocampal neuronal cell line HT-22 (Huiying, China) and the murine Schwann cell line RSC-96 (ATCC, USA) were cultured in Dulbecco’s modified Eagle’s medium (DMEM) with 10% FBS. RSC-96 cells were treated with capsaicin (1 μM, M3422, AbMole BioScience, USA) for 48 h, followed by culture in serum-free DMEM for an additional 48 h. The exRNA from the medium was collected for subsequent applications. The cells were then treated with the exRNAs released from RSC-96 cells.

#### Quantification of exRNAs and dsRNAs

The exRNA concentration of serum was quantified using RNA Select Green Fluorescent Cell Stain (S32703, Molecular Probes, USA). The level of dsRNAs in serum was assessed using the dsRNA Elisa Kit (Bunsenbio, Tianjin, China), which measures the optical density value of dsRNA. For frozen brain and sciatic nerve sections or cells, staining was performed for 20 min at 37 °C, followed by washing with PBS. The experiment was conducted under strict RNase-free conditions.

#### Stereotaxic surgery and intrahippocampal microinjection

The adeno-associated virus (AAV) rAV-hSyn-mCherry-5’miR-30a-shRNA-3’miR-30a-WPREs (Shumi Technology, Wuhan, China) was used for TLR3 neural-specific knockdown. The transfection reagent (Engreen, Beijing, China) was used to dilute the primary solution of AAV-TLR3 (AAV^+^) and AAV-control (AAV^−^). A stereotaxic apparatus (RWD Life Science, Shenzhen, China) was used for the injection of the hippocampus. AAV solution (0.2 µl) was slowly injected into the hippocampus by a NanoFil needle, a NanoFil syringe and a MicroSyringe Pump Controller (World Precision Instruments, Sarasota, FL, USA). The coordinates are antero-posterior = −2.00 mm, medio-lateral = ±1.8 mm and dorso-ventral= −1.7 mm. Bilateral intrahippocampal injection of AAV-TLR3 was the same as AAV^−^ control.

#### Real-time qRT‒PCR

Total RNA was separated through total RNA extraction TRIzol reagent (Invitrogen Life Technologies) and centrifuge columns. The progress was conducted in the Mastercyclerep realplex PCR system (Eppendorf, Hauppauge, NY). Primer sequences for qRT‒PCR are listed in Supplementary Table 1.

#### Western blot analysis

Equal amounts of protein (10 μg) from tissues or cells were separated using SDS‒PAGE and transferred onto a polyvinylidene fluoride (PVDF) membrane (Millipore). Next, the membranes were subjected to overnight incubation at 4 °C with primary antibodies. Subsequently, the membranes were incubated with secondary antibodies. The results were shown using the detection reagent and analyzed using ImageJ. α-Tubulin or GAPDH was employed as the control.

#### TUNEL staining

Apoptosis of the brain tissue was assessed using TUNEL staining with a cell death detection kit (C1091, Beyotime). The count of cells exhibiting green fluorescence was quantified in four microscopic fields within the hippocampal region. Subsequently, the percentage of nuclei displaying TUNEL-positive signals was computed.

#### Golgi staining

The brains were subjected to Golgi-Cox staining using the Golgi Stain kit (Neurotechnologies, Columbia). Brain tissue sections were obtained at a thickness of 150 μm using a vibrating microtome (VT1000; Leica Microsystems, Germany). The staining of the sections followed the instruction manual. Pyramidal neurons in the CA1 region were observed and screened using a microscope with an oil-immersion objective at magnifications of 320 and 3100x (Zeiss AX10 imager A2/AX10 cam HRC, Germany). Neuron J (NIH, Bethesda, MD) was utilized for the tracing and analysis of neurons.

### Statistical analysis

The data are presented as the means ± SEMs. Statistical analysis was conducted using GraphPad Prism 9 software. Data from WB, immunofluorescence, and Golgi staining were analyzed using NIH ImageJ software. When comparing two groups and the data passed the normality test, a *t*-test was used. If the data did not pass the normality test, a nonparametric test, such as the Mann‒Whitney test, was used. When comparing four groups and there were multiple factors in the behavioral results, a two-way ANOVA was used. Otherwise, one-way ANOVA was employed. Statistical significance was defined as a probability value less than 0.05 (*P* < 0.05).

### Supplementary information

Supplementary Materials

## Data Availability

The datasets for the current study are available from the corresponding author upon reasonable request.
