# Supplementary material for: Extracellular RNAs-TLR3 signaling contributes to cognitive impairment after chronic neuropathic pain in mice
Source: Signal Transduct Target Ther. 2023 Aug 7;8:292. doi: 10.1038/s41392-023-01543-z (PMC10404588; doi:10.1038/s41392-023-01543-z)
Supplement: Supplementary file 1 — Supplementary Materials [file 41392_2023_1543_MOESM1_ESM.docx]

Supplementary Materials for

Extracellular RNAs-TLR3 signaling contributes to cognitive impairment after chronic neuropathic pain in mice

Xueying Zhang^1, 2, #,^ Rui Gao^1, 2, #^, Changteng Zhang^1, 2, #^, Yi Teng^1, 2^, Hai Chen^3^, Qi Li^1, 2^, Changliang Liu^1, 2^, Jiahui Wu^1, 2^, Liuxing Wei^1, 2^, Liyun Deng^1, 2^, Lining Wu^1, 2^, Shixin Ye-Lehmann^4^, Xiaobo Mao^5^, Jin Liu^1, 2^, Tao Zhu^1, 2, *^, Chan Chen^1, 2, *^.

Correspondence to: xychenchan@gmail.com/chenchan@scu.edu.cn

**This PDF file includes:**

Figures. S1 to S11

Tables S1


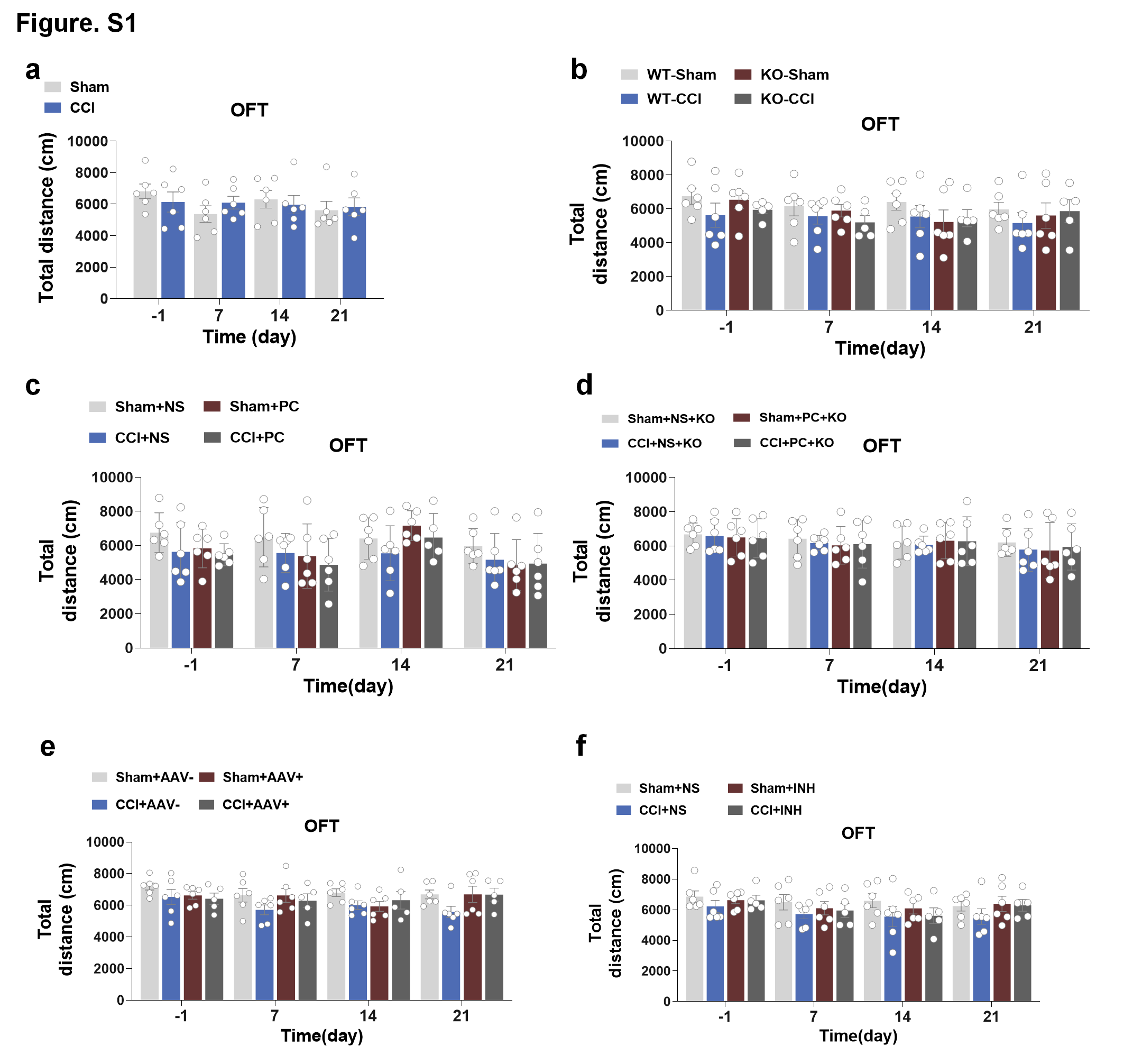


**Figure. S1.** OFT results of all parts of experiments. **a** OFT was used to examine locomotor activity between Sham group and CCI group. **b** The compare of OFT distance between WT-Sham, WT-CCI, KO-Sham and KO-CCI group. **c** The compare of OFT distance between Sham+NS, CCI+NS, Sham+PC, CCI+PC group. **d** The compare of OFT distance between Sham+NS+KO, CCI+NS+KO, Sham+PC+KO, CCI+PC+KO group. **e** The compare of OFT distance between Sham+AAV^-^, CCI+AAV^-^, Sham+AAV^+^, CCI+AAV^+^ group. **f** The compare of OFT distance between Sham+NS, CCI+NS, Sham+INH, CCI+INH group.


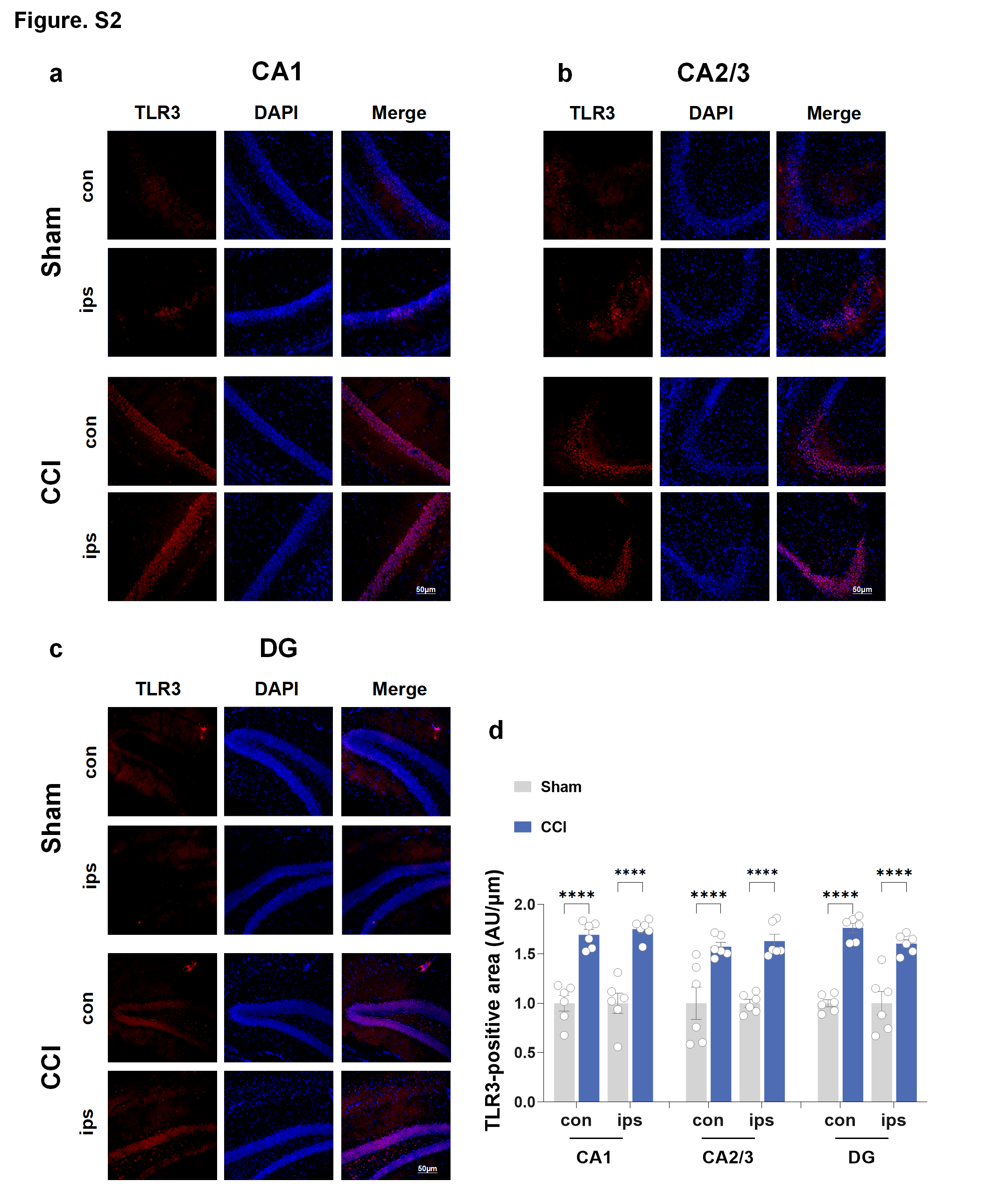


**Figure. S2.** TLR3 increased in the CA1, CA3, and DG region of both the ipsilateral and contralateral hippocampus after CCI. **a-d** The images of TLR3 expression in the CA1, CA3, and DG region of both the ipsilateral and contralateral hippocampus from the sham group and CCI group (*****P* <0.0001).


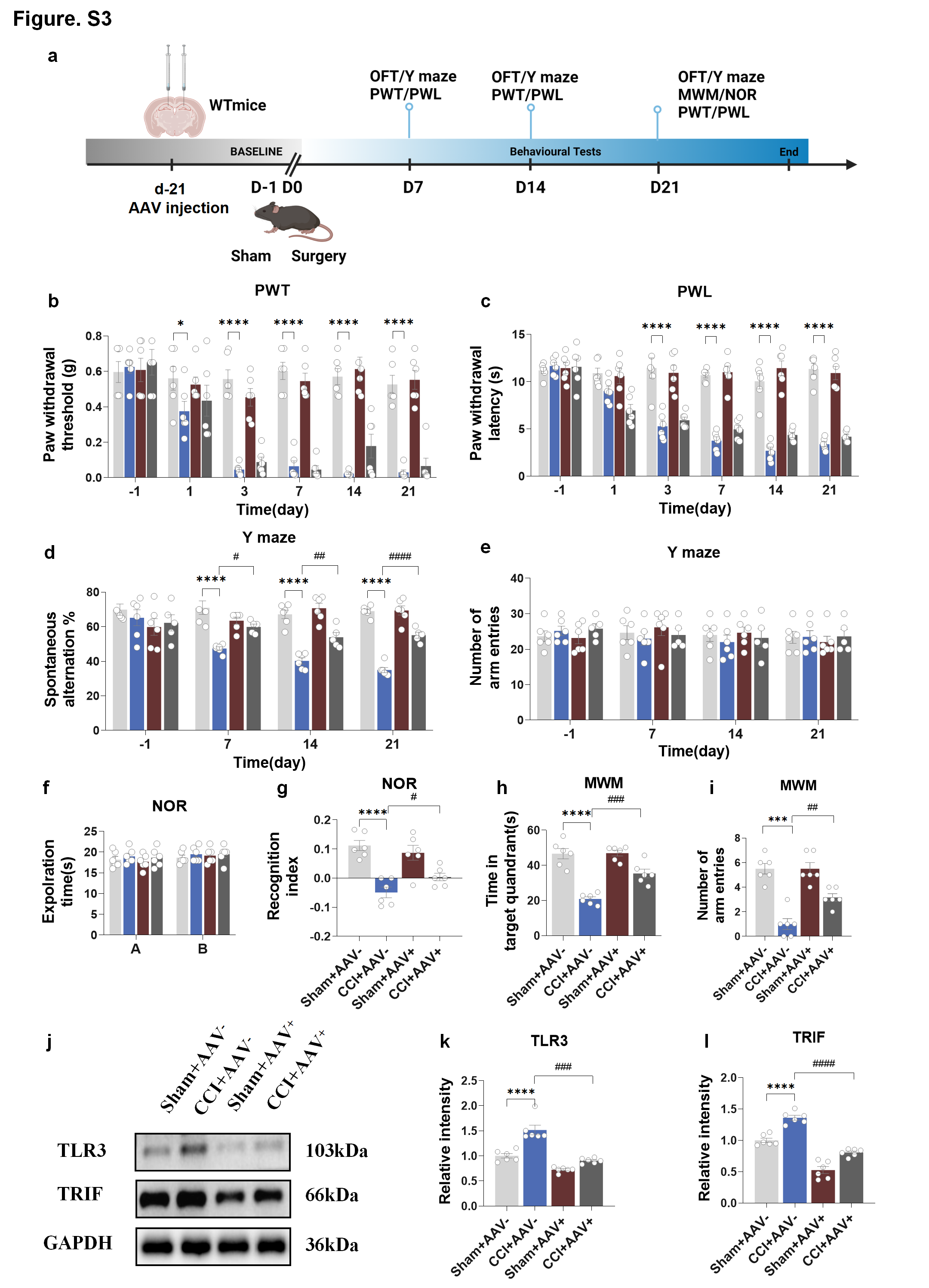


**Figure. S3** Effects of neuronal specific knock-down TLR3 on the chronic constriction injury-induced cognitive decline in mice. **a** The flowchart of this experiment. **b, c** Mechanical allodynia was evidenced by reductions in PWT, whereas thermal hyperalgesia was demonstrated by the decreases in PWL. **d, e** In the Y maze test, number of arm entries and the spontaneous alternation were analyzed. **f, g** In the NOR test, the investigation time of objects was recorded in the training and test periods, and the discrimination index was calculated in the test period. **h, i** In the MWM test, the time in target quadrant and numbers of cross the platform at the testing day were record. **j-l** The WB results also showed that the expression of TLR3 and TRIF in the hippocampus was decreased in the CCI+AAV^+^ group compared with the CCI+AAV^-^ group. Data are presented as mean ± SEM (n = 6 per group). ^*^, Sham+AAV^-^ vs. CC+ AAV^-^; ^#^, CCI+ AAV^-^ vs. CCI+ AAV^+^, ^*^*P* < 0.05, ^**^*P* <0.01, ^***^*P* <0.001, ^****^*P* <0.0001; ^#^*P* < 0.05, ^##^*P* <0.01, ^###^*P* <0.001, ^####^*P* <0.0001.


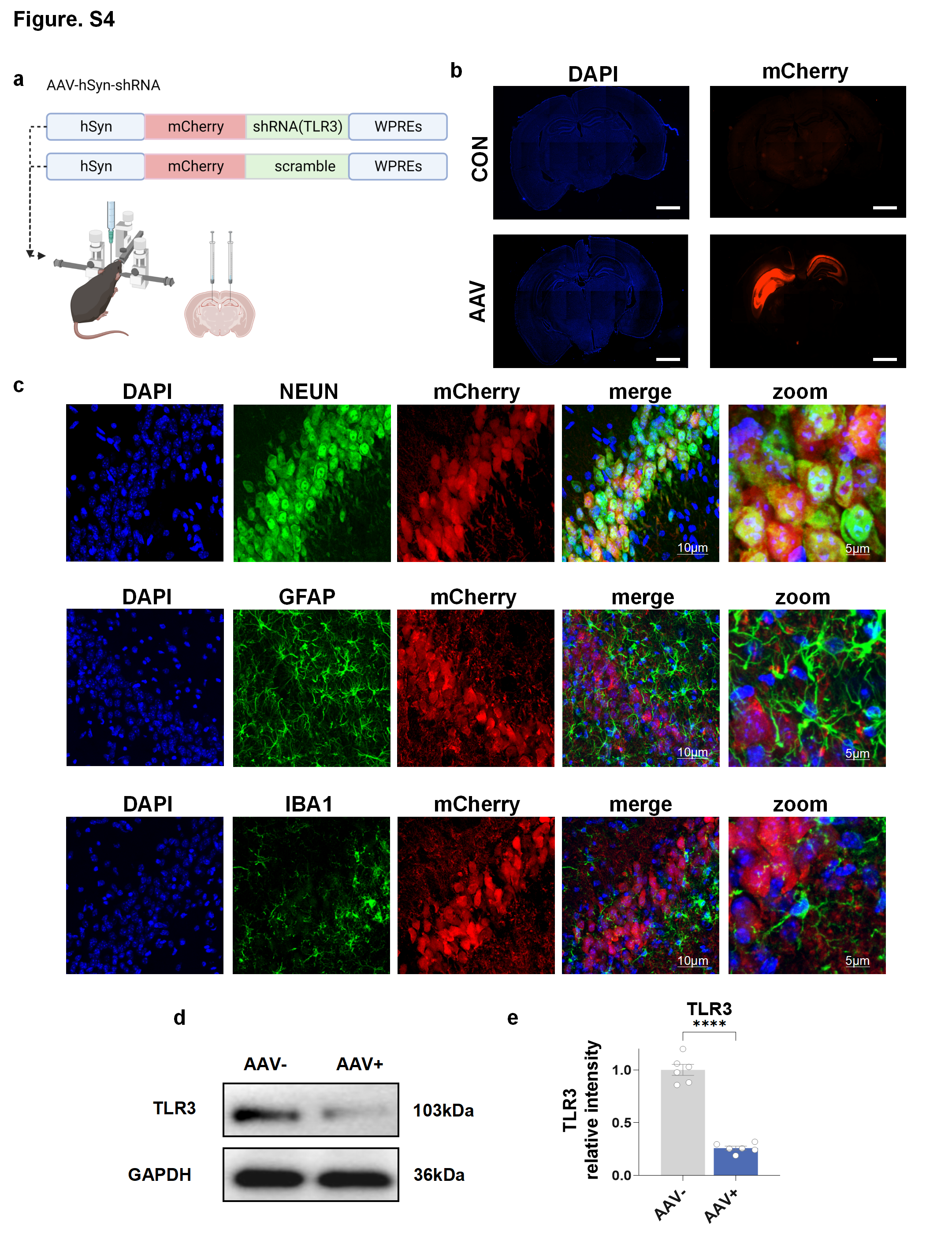


Figure. S4 Effects of neuronal specific knock-down TLR3 AAV on the expression of TLR3 in hippocampus. a The model diagram of AAV. b Fluorescent pictures of 21 days after AAV injection. Scale bar = 500 μm. c Immunofluorescent staining results of the Cherry expression on neuron, microglia and astroglia. Scale bar = 10 μm (DAPI, NEUN/GFAP/IBA, mCherry and merge). Scale bar = 5 μm (zoom). d, e WB results showed TLR3 was decreased significantly in hippocampus of AAV^+^ group. ^****^*P* <0.0001.


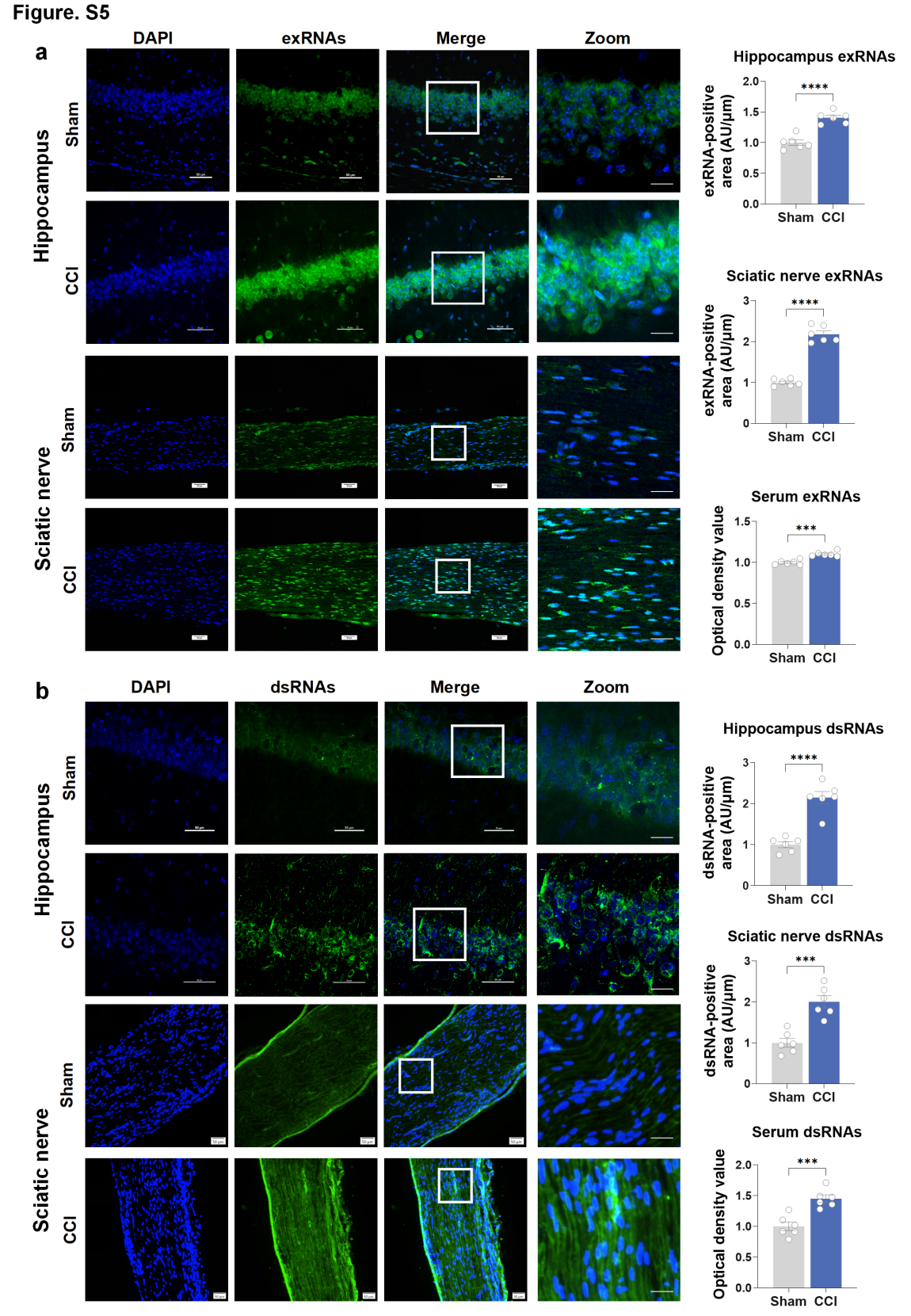


**Figure. S5** Increased exRNAs/dsRNAs levels were detected after chronic constriction injury. **a** Immunofluorescent staining of the exRNAs expression in the hippocampus, sciatic nerve and serium on day 21 after sham surgery and CCI. **b** Immunofluorescent staining of the dsRNAs expression in the hippocampus and sciatic nerve and Elisa result of dsRNAs in serum on day 21 after sham surgery and CCI. Scale bar = 50 μm (DAPI, exRNAs/dsRNAs and merge). Scale bar = 10 μm (zoom). Data are presented as mean ± SEM (n = 6 per group). ^***^*P* <0.001, ^****^*P* <0.0001.


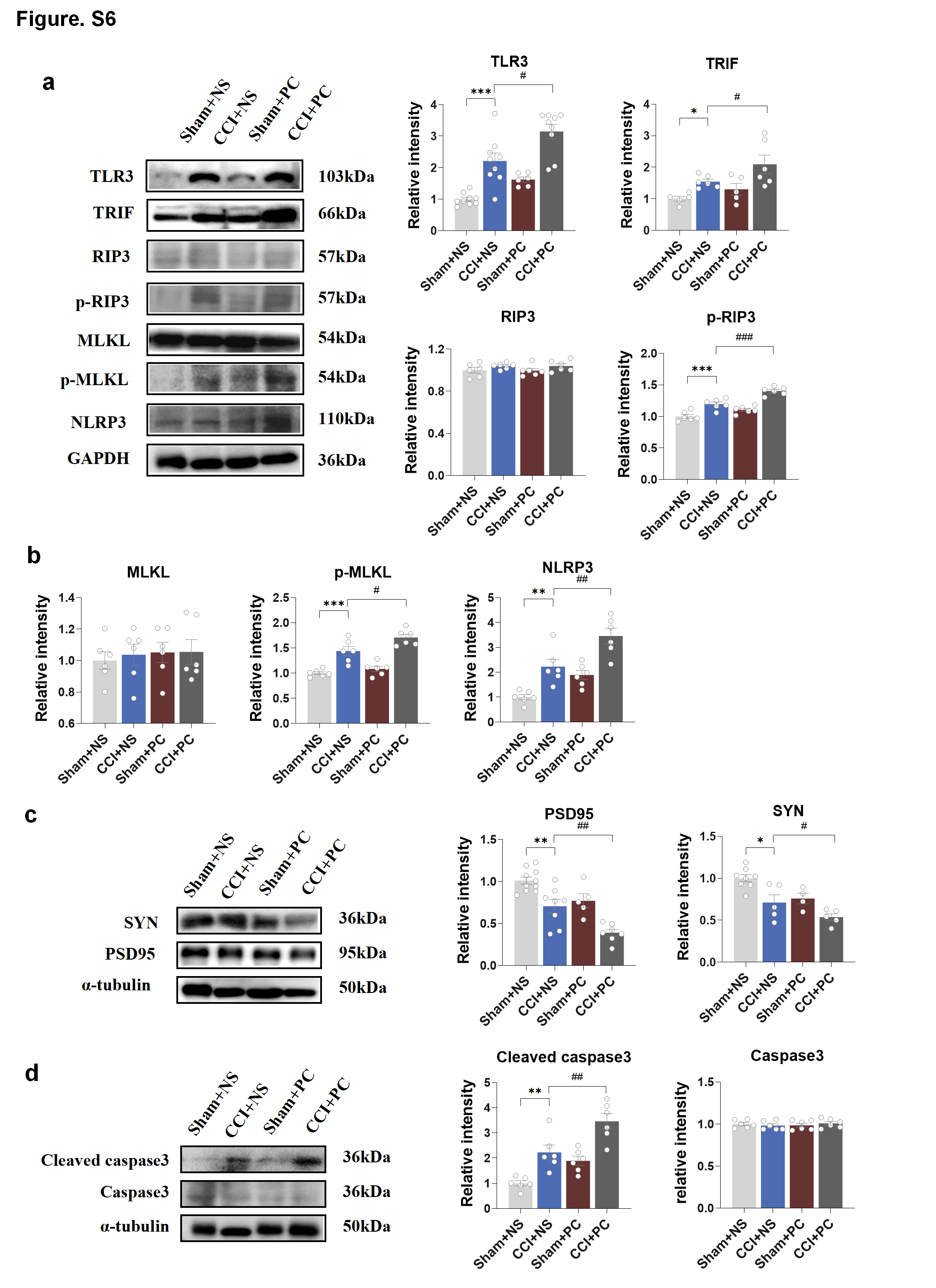


**Figure. S6** Poly (I:C) exacerbate cognitive impairment induced by chronic constriction injury. a, b The effect of Poly (I:C) on the TLR3, TRIF, RIP3, p-RIP3, MLKL, p-MLKL and NLRP3 proteins expression in the hippocampus on day 21 after CCI and sham surgery. c The SYN and PSD95 proteins expression on day 21 after CCI and sham surgery. d The protein level of caspase3/cleaved caspase3 apoptosis signal pathway on day 21 after CCI and sham surgery. Data are presented as mean ± SEM (n = 6 per group). ^*^, Sham+NS. vs. CC+NS.; I; ^#^, CCI+PC vs. CCI+PC. NS.,nornal saline; PC, Poly (I:C). ^*^*P* < 0.05, ^**^*P* <0.01, ^***^*P* <0.001; ^#^*P* < 0.05, ^##^*P* <0.01, ^###^*P* <0.001.


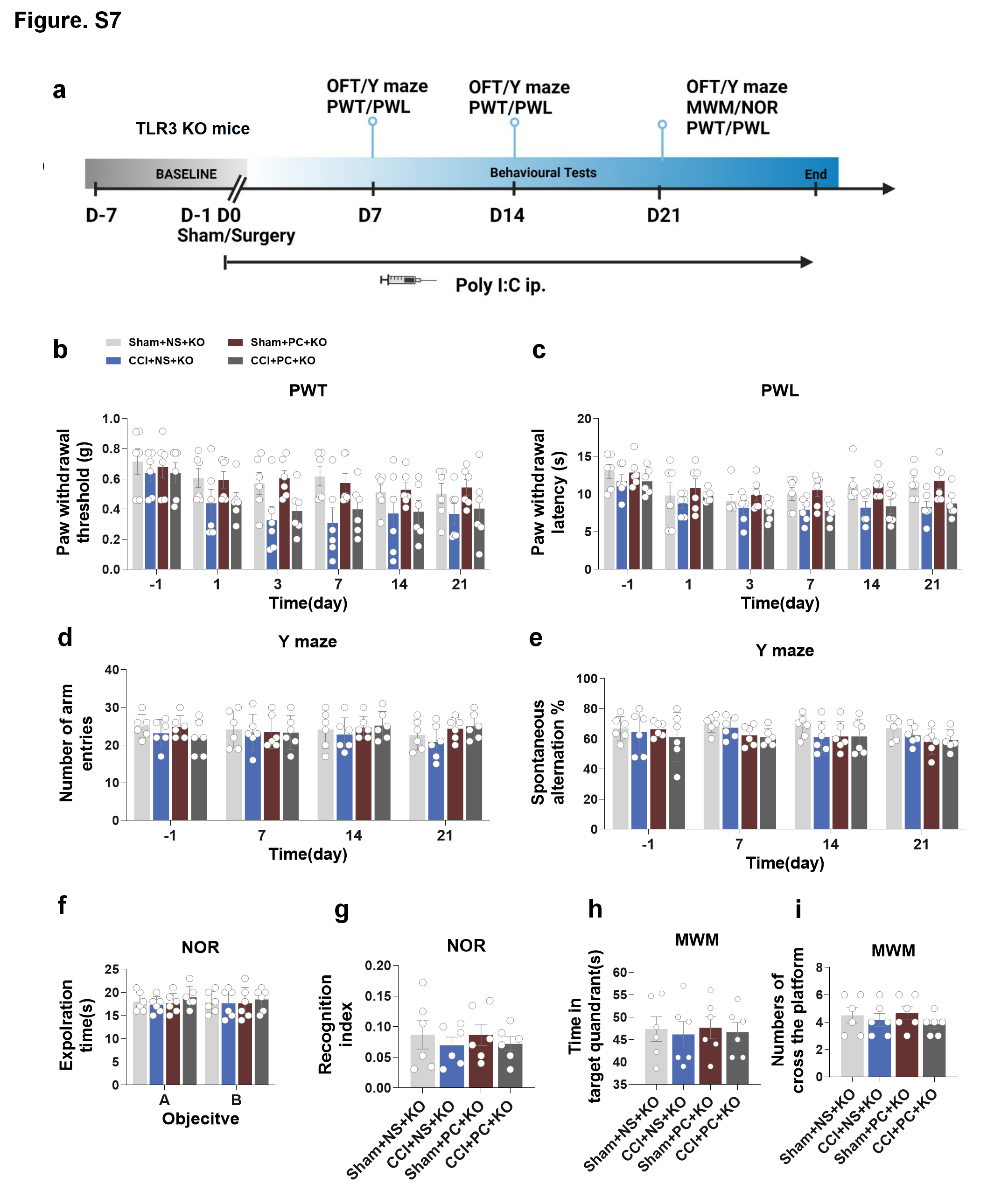


**Figure. S7** The effects of Poly (I:C) on TLR3 KO mice. a The flowchart of this experiment. b, c Mechanical allodynia was evidenced by reductions in PWT, whereas thermal hyperalgesia was demonstrated by the decreases in PWL. d, e In the Y maze test, number of arm entries and the spontaneous alternation were analyzed. f, g In the NOR test, the investigation time of objects was recorded in the training and test periods, and the discrimination index was calculated in the test period. h, i In the MWM test, the time in target quadrant and numbers of cross the platform at the testing day were record. Data are presented as mean ± SEM (n = 6 per group).


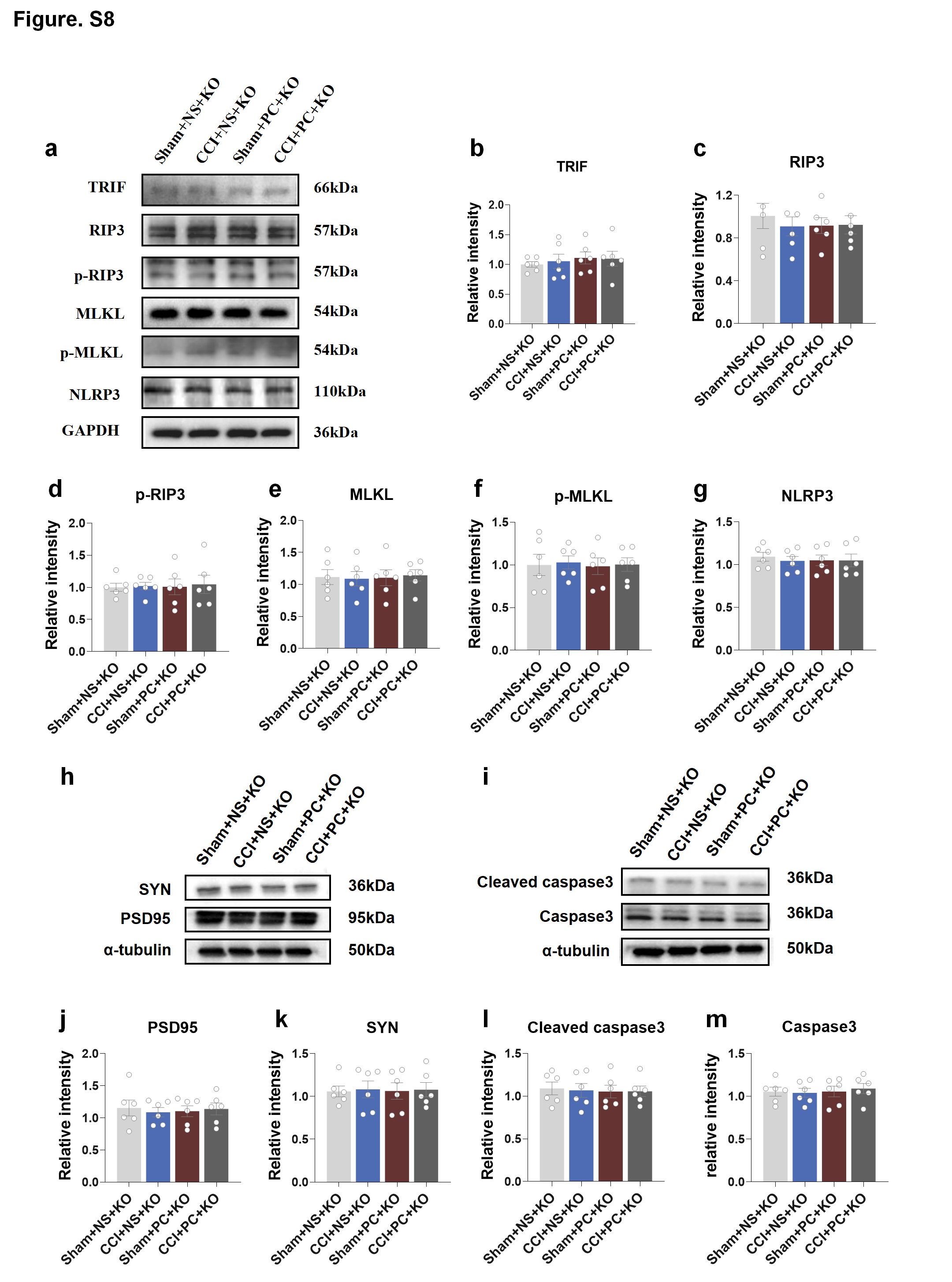


**Figure. S8** The effects of Poly (I:C) on TLR3 down-stream pathway of TLR3 KO mice. a-g The effect of Poly (I:C) on the TLR3, TRIF, RIP3, p-RIP3, MLKL, p-MLKL and NLRP3 proteins expression in the hippocampus on day 21 after CCI and sham surgery of TLR3 KO mice. h, j, k The SYN and PSD95 proteins expression on day 21 after CCI and sham surgery. i, l, m The protein level of caspase3/cleaved caspase3 apoptosis signal pathway on day 21 after CCI and sham surgery. Data are presented as mean ± SEM (n = 6 per group).


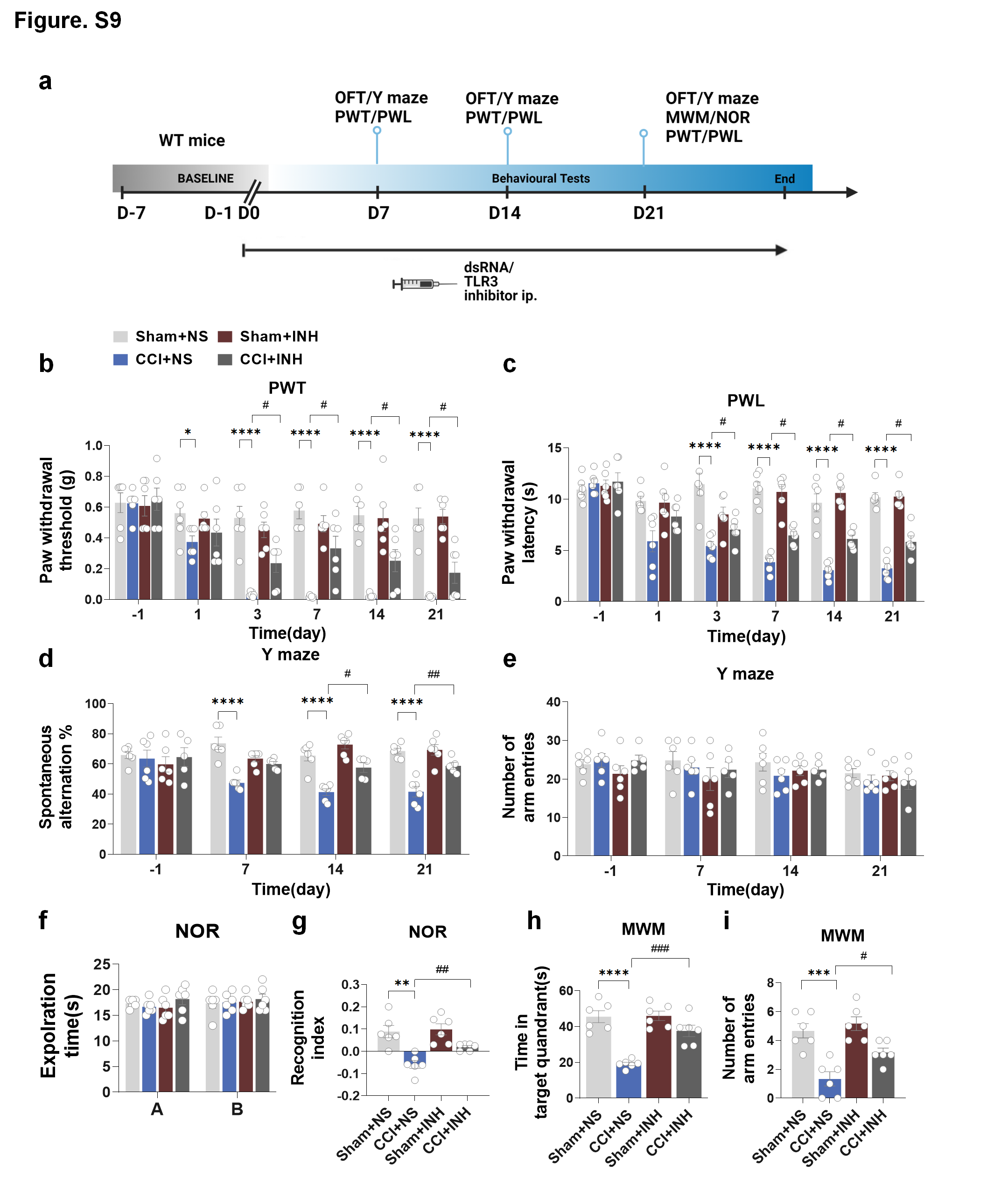


**Figure. S9** Effects of dsRNA/TLR3 inhibitor on the chronic constriction injury-induced nociceptive hypersensitivity and cognitive decline in mice. **a** The flowchart of this experiment. **b, c** Mechanical allodynia was evidenced by reductions in PWT, whereas thermal hyperalgesia was demonstrated by the decreases in PWL. **d, e** In the Y maze test, number of arm entries and the spontaneous alternation were analyzed. **f, g** In the NOR test, the investigation time of objects was recorded in the training and test periods, and the discrimination index was calculated in the test period. **h, i** In the MWM test, the time in target quadrant and numbers of cross the platform at the testing day were record. Data are presented as mean ± SEM (n = 6 per group). ^*^, Sham+NS vs. CC+NS; I; ^#^, CCI+INH vs. CCI+INH, NS, nornal saline; INH, inhibitor. ^*^*P* < 0.05, ^**^*P* <0.01, ^***^*P* <0.001; ^#^*P* < 0.05, ^##^*P* <0.01, ^###^*P* <0.001.


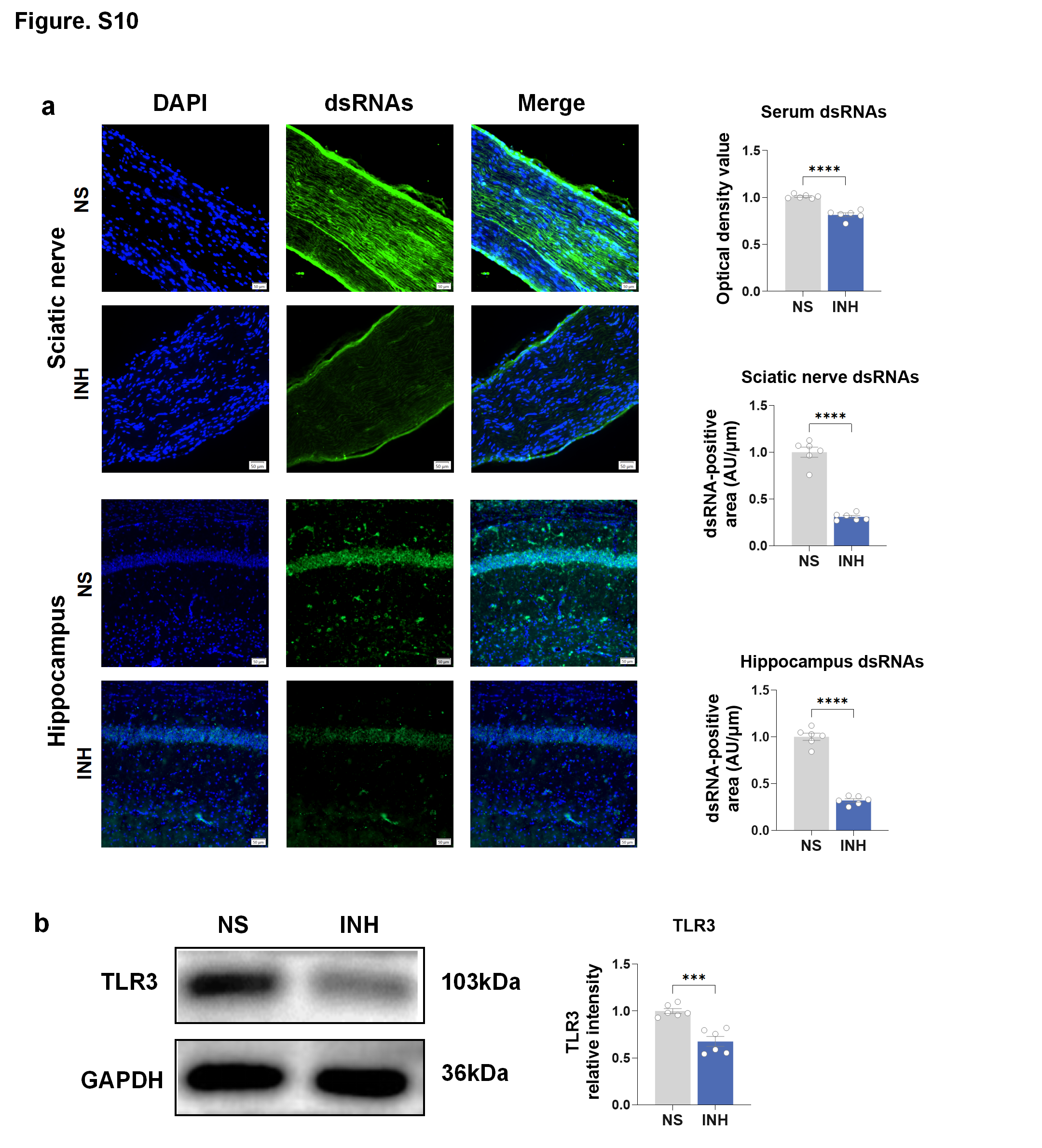


**Figure. S10** The effects of dsRNAs/TLR3 inhibitor on expression of dsRNAs and TLR3. a The effects of dsRNAs/TLR3 inhibitor on expression of dsRNAs in hippocampus, sciatic nerve and serum. b WB results of TLR3 protein in hippocampus. Scale bar = 50 μm. Data are presented as mean ± SEM (n = 6 per group). ^***^*P* <0.001, ^****^*P* <0.0001.


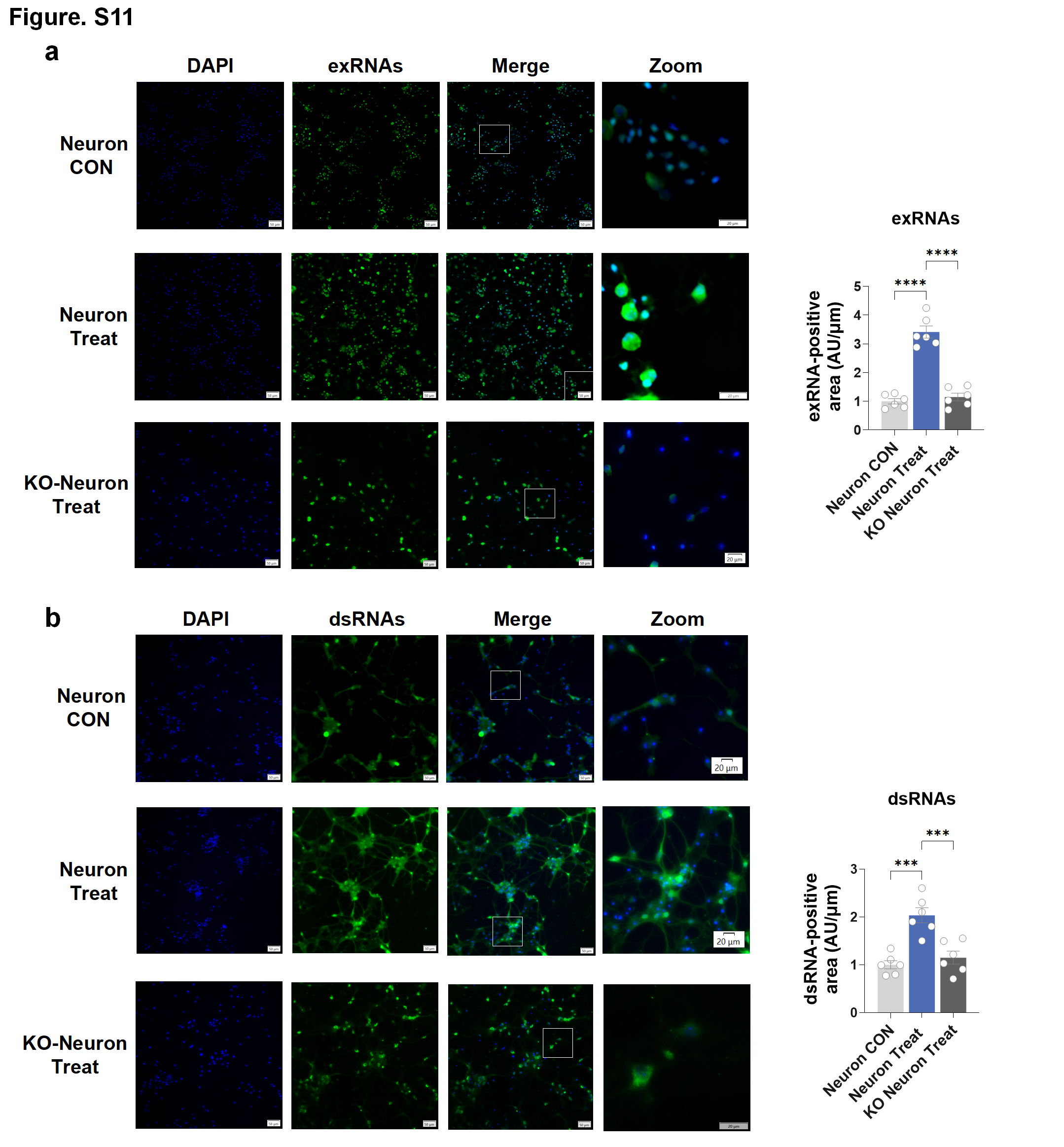


**Figure. S11** exRNAs was upregulated in primary neuron after RNAs treatment. **a** The effect of exogenous exRNAs/dsRNAs derived from RSC96 cells on the exRNAs for the primary cultured neurons. **b** The effect of exogenous exRNAs/dsRNAs derived from RSC96 cells on the dsRNA for the primary cultured neurons. Data are presented as mean ± SEM (n = 6 per group). ^*^, CON vs. Treat. CON, control groups without exogenous exRNAs/dsRNAs treated; treat, groups with exogenous exRNAs/dsRNA treated. ^****^*P* <0.001.

Table S1.

PCR primers used in this study.

| Gene | Forward (5′ → 3′) | Reverse (3′ → 5′) |
| --- | --- | --- |
| TRIF | CCT GAG CCT GCA TCA AAT C | CCA CCT TTC TGG CGA AGA |
| NLRP3 | TGG GTT CTG GTC AGA CAC GAG | ACA CGT GTC ATT CCA CTC TGG |
| IL-1β | TGC CAC CTT TTG ACA GTG ATG | AGG CCA CAG GTA TTT TGT CGT |
| TNF-α | CTG TGA AGG GAA TGG GTG TT | CAG GGA AGA ATC TGG AAA GGT C |
